# The effect of an educational intervention for university students on violence against the elderly using gamification: a non-randomized clinical trial*

**DOI:** 10.1590/1518-8345.7874.4662

**Published:** 2025-08-18

**Authors:** Juliana Ribeiro da Silva Vernasque, Miriam Fernanda Sanches Alarcon, Daiana Bonfim, Paula Sales Rodrigues, Eduardo Federighi Baisi Chagas, Maria José Sanches Marin

**Affiliations:** 1 Faculdade de Medicina de Marília, Educação em Ciências da Saúde, Marília, SP, Brazil.; 2 Universidade Estadual Paulista, Departamento de Enfermagem, Botucatu, SP, Brazil.; 3 Scholarship holder at the Coordenação de Aperfeiçoamento de Pessoal de Nível Superior (CAPES), Brazil.; 4 Universidade Estadual do Norte do Paraná, Departamento de Enfermagem, Bandeirantes, PR, Brazil.; 5 Faculdade Israelita de Ciências da Saúde Albert Einstein, Centro de Estudos, Pesquisa e Prática APS e Redes, São Paulo, SP, Brazil.; 6 Faculdade de Medicina de Marília, Estatística, Marília, SP, Brazil.; 7 Faculdade de Medicina de Marília, Enfermagem em Saúde Coletiva, Marília, SP, Brazil.

**Keywords:** Aged, Violence, Universities, Elder Abuse, Attitude, Knowledge

## Abstract

to evaluate the impact of a gamified educational intervention on violence against the elderly on the knowledge, perceptions and attitudes of university students.

a non-randomized clinical trial involving 44 university students from the health sciences, humanities and exact sciences, with 22 in the intervention group and 22 in the control group. The intervention group participated in hybrid activities on gamification applied to the topic of violence against the elderly. The evaluation was conducted using thematic cases validated by experts, with statistical analysis using Chi-square and Student’s t tests.

the intervention promoted significant differences between the groups in attitudes and perceptions about violence against the elderly. Students in the intervention group were more likely to report cases of neglect and were more aware of how to prevent abuse in public spaces.

the gamified intervention was effective in stimulating protective attitudes and ethical perceptions of violence against the elderly, but there was no significant association with the knowledge variable. There is a need for more comprehensive studies that can provide complementary evidence to the results of this study.

## Highlights

**(1)** University students are more likely to report family neglect. **(2)** Increased awareness of the rights of older people in public spaces. **(3)** Intervention promotes practical awareness of elder abuse. **(4)** Innovative approaches are effective in training more empathetic professionals. **(5)** Gamified intervention promotes protective attitudes towards older people.

## Introduction

Violence against older people, characterized by acts or omissions that cause harm or suffering to this population, is a globally acknowledged problem^(1)^. It is estimated that for every case recorded, another five go unreported, highlighting the underreporting and invisibility of the problem^(2)^.

Violence against the elderly takes many forms - physical, psychological, sexual and financial abuse as well as neglect - each of which has severe impacts and requires a multi-sectoral approach to be identified and counteracted. Notably, the majority of perpetrators are family members, which makes reporting and intervention difficult^(3)^. Recognition of violence against older people is still late and inadequate in the various fields of knowledge, and it is considered an “underfunded, under-researched and under-recognized” problem^(4)^.

Identifying and reporting situations of violence against older people is a complex and cautious process. In addition, there is a lack of knowledge among different sectors of society and among older people themselves about their rights and the meaning of violence^(5)^.

Studies show that educational interventions can be effective in preventing elder abuse. A cross-sectional study in the United States of America (USA) of 2,150 elderly Korean Americans analyzed the prevalence and factors associated with physical, emotional, and financial abuse and concluded that preventive strategies and contextualized educational interventions are needed^(6)^. In Israel, a study of 145 orthopedic surgeons found that knowledge and more positive attitudes were associated with increased identification and reporting of cases, highlighting the importance of training in the recognition and management of elder abuse from the beginning of their careers^(7)^. In addition, online training at a university in the United States^(8)^ was shown to significantly improve students’ knowledge of how to interact appropriately with elderly victims of abuse. Similar results were found among Iranian nurses^(9)^, where higher levels of education were associated with better care practices, although barriers such as workload and lack of training still hinder the application of knowledge.

In this context, it is understood that interdisciplinary educational actions that promote meaningful learning are essential to stimulate not only knowledge, but also perceptions and protective attitudes towards the elderly population. By involving students in gamified training that integrates values and practical skills, it is hoped that a deeper understanding and attitudes of respect and protection, which are necessary to address this complex issue, will be fostered.

Gamification is the use of game design elements and techniques in education, health care and business. In the educational context, gamification, especially when combined with collaborative learning, contributes to the development of transversal skills such as creativity, problem solving and teamwork as well as improves academic performance and student motivation^(10)^.

Thus, the research question is: Do young university students trained to recognize violence against the elderly through gamification improve their knowledge, attitudes, and perceptions, resulting in greater responsibility than untrained university students?

The purpose of this study was to evaluate the impact of a gamified educational intervention on violence against the elderly on the knowledge, perceptions, and attitudes of university students.

## Method

### Study design

This is a non-randomized clinical trial. In order to maintain methodological rigor, the Standards for QUality Improvement Reporting Excellence in Education (SQUIRE 2.0) protocol was used as a support tool for the development of the study^(11)^.

The non-randomized design was selected due to the nature of the study and the characteristics of the application context^(12)^. Given the challenges associated with randomizing the allocation of participants into intervention groups, the study opted to involve university students who were available and willing to participate, thus circumventing the need for random division between groups. This methodological approach permitted the implementation of an educational intervention within a real-world setting, thereby facilitating a more flexible and pragmatic analysis of the intervention’s impact on the participants’ knowledge, perceptions, and attitudes.

### Study site

The study site was a medium-sized municipality in the center-west of São Paulo state (Brazil), with an estimated population of 235,234 inhabitants^(13)^. This municipality has a large complex of higher education institutions (HEIs), both public and private, which offer programs in different fields of knowledge. For this study, four HEIs were included, two public and two private, in order to gather university students from the health sciences, exact sciences and humanities.

### Period

The study was conducted from December 2022 to April 2024.

### Population

Participants in this study were university students from the humanities, exact and health sciences, who were selected non-randomly given the context and nature of the educational intervention. Two groups were formed: the first (intervention group) consisted of young university students who participated in the educational intervention; and the second (control group) consisted of young university students who did not participate in the educational intervention. During the study period, these participants maintained their regular academic activities, with no involvement in activities related to the intervention topic and no specific changes in their academic routine.

### Selection criteria

The intervention group was recruited in December 2022 to participate for one year, until December 2023. Initially, 40 young university students were gathered to select the participants (intervention group): students from the programs of Nursing, Medicine, Law, Occupational Therapy, Physical Therapy, Civil Engineering, Psychology and Pedagogy. The students were selected according to their interest in participating in the educational intervention. Twenty-two students from the following programs participated in the whole process: Nursing, Medicine, Law, Occupational Therapy and Physical Therapy.

Members of the control group were selected by convenience. They were recruited in March 2024 and did not receive the intervention. In order to form the groups, invitations were made in collaboration with teachers and program coordinators of the participating institutions. The design of the intervention was controlled; the groups were formed by pairing, thus ensuring a rigorous and open composition^(12)^.

The inclusion criteria for the study were as follows: to be a university student; to be enrolled at one of the four participating universities; and, in the case of the intervention group, to be available to participate in educational activities for one year. The following were excluded from the study: university students who were not enrolled in programs in the health sciences, humanities, or exact sciences; students who did not have access to digital devices or the Internet; and individuals who did not complete the data collection instrument.

### Intervention

The educational intervention took place over the course of a year, with four face-to-face meetings interspersed with online meetings, for a total of 50 hours. The educational process used gamification through lectures, classroom sessions including discussions, and workshops that encouraged students to reflect on violence against the elderly based on their own experiences, as well as on the possibilities of developing games to promote elderly health and prevent violence. The activities were supported by teachers and researchers on violence against the elderly and by a game specialist.

It is understood that the use of information technologies in educational processes, in all fields of knowledge, can enhance meaningful learning and contribute to building practical knowledge and changing attitudes.

In the first face-to-face meeting, a presentation was given on “Violence against the elderly”, covering key concepts, epidemiological aspects and the results of studies carried out in the community itself, highlighting the problems faced by this section of the population^(14)^.

Next, a dialogic presentation was given on the principles and stages of gamification, highlighting the intersection between the two topics of gamification and violence against the elderly.

In the work dynamics, the students were divided into groups based on affinities to develop game proposals on the topic of violence against the elderly. At each new meeting, the groups presented the progress of their work, followed by a space for discussion, so that the other participants could analyze and evaluate the projects under development and make suggestions for improving the proposals. In this way, the proposals of each group were built and refined within the small groups and with the contribution of all the participants.

In the end, six projects were developed using gamification, which allowed the students involved to remain engaged throughout the period, both in relation to the topic and in the actual construction of a project that could have a practical application in society.

### Study variables

To characterize the sample, data were collected on age, gender, and degree program. To evaluate the effectiveness of the intervention, the percentage of correct answers to the proposed cases was taken as the primary outcome. Five cases were prepared, each intended to address a specific form of violence against the elderly, allowing for a broad analysis of the different types of abuse.

A Likert scale was used to validate the cases by the reviewers, allowing the referees to express the degree of agreement with the suitability of each question for the intended evaluation.

This process was designed to ensure that the questions accurately captured the students’ perceptions, technical knowledge and attitudes towards different situations of violence faced by older people in different contexts. In addition, space was provided for suggestions and opinions on specific content if deemed necessary by the evaluator. The validation of the content of the cases was carried out with the participation of 11 invited referees with expertise either in the field of geriatrics and gerontology or in learning assessment processes, who signed an informed consent form.

Regarding the profiles of the referees: nine women and two men; average age: 53 years; professional training: three with a degree in nursing, four in medicine, two in psychology, one in pharmacy and one in social work. The average length of training in years was 29.54, and the average length of time in their current job was 26.54 years. Validation took place at two different times, considering that the evaluators made pertinent suggestions that were added to the evaluation instrument.

The Content Validity Coefficient (CVC)^(15)^ was used to analyze the level of agreement among the referees. CVC is a recommended indicator for calculating the level of agreement by calculating the mean value assigned by the referees to each item in the instrument, expressed in percentages. We calculated the individual CVC for each question and the mean of all of them for the overall CVC, and items with percentages equal to or greater than 80% were considered valid^(16)^. CVC was calculated for the instrument with the five cases and the six questions respectively, and it was 0.94 for both.

The validated cases were entered into a Google Form and then sent to participants via WhatsApp, both in the intervention and control groups. Case 1 focuses on family neglect, assessing students’ ability to identify signs of carelessness and omission in elder care. Case 2 focuses on financial violence, assessing students’ perceptions of economic exploitation and inappropriate control of the elder’s assets. Case 3 focuses on physical violence in a long-term care facility and seeks to assess recognition of signs of physical aggression. Case 4 deals with disregard for the rights of older people on public transport, encouraging students to reflect on attitudes of citizenship and respect. Finally, Case 5 explores sexual violence, focusing on recognizing abuse and the consequences of abusive relationships.

For each case, there were six questions to assess the participants’ knowledge, perceptions, and attitudes. Questions one and six were designed to assess students’ perceptions of violence against the elderly in society; questions two and three were intended to measure knowledge; and questions four and five were aimed at assessing attitudes. Each question was answered with the options “yes”, “no”, “I can’t say for sure”, “not enough knowledge”, and “o definite position”. However, only the sum of the correct answers was considered when calculating the percentage of correct answers.

### Data analysis

The qualitative variables are described by absolute (N) and relative (%) frequency distribution. The Chi-square proportion test was used to analyze differences in frequency distribution. The Chi-square association test was applied to analyze the relationship between qualitative variables and groups. Student’s t-test for independent samples was used to compare means after checking the homogeneity of variances using Levene’s test. The significance level adopted was 5%, and the data were analyzed using the SPSS software (version 27.0).

### Ethical aspects

The study was approved by the Research Ethics Committee (CEP) under Report No. 5.144.186, and all participants signed an informed consent form.

## Results

There were 22 students in the intervention group and 22 in the control group. The mean age in the intervention group was 23 years, with a standard deviation of 2.11; the mean age in the control group was 24.04 years, with a standard deviation of 2.11.

The intervention and control groups were homogeneous regarding gender and degree program (Table 1).

**Table 1-:** Distribution of participants, intervention and control groups, by gender and degree program (n = 44). Marília, SP, Brazil, 2024

		**Group**				**Total**		*p* -value a*	***p* -value b** ^†^
		**Intervention (n=22)**		**Control (n=22)**					
		**N**	**%**	**N**	**%**	**N**	**%**		
Gender	Male	5	22.7%	5	22.7%	10	22.7%	<0.001 ^‡^	0.999
	Female	17	77.3%	17	77.3%	34	77.3%		
Degree Program	Medicine	10	45.5%	12	54.5%	22	50.0%	<0.001 ^‡^	0.904
	Nursing	8	36.4%	5	22.7%	13	29.5%		
	Occupational Therapy	1	4.5%	1	4.5%	2	4.5%		
	Law	2	9.1%	2	9.1%	4	9.1%		
	Physical Therapy	1	4.5%	2	9.1%	3	6.8%		

*p-value a = p-value calculated by the Chi-square test for proportion. This test checks whether there is a difference in the proportion distribution in the response categories as a whole; ^†^p-value b = p-value calculated by the Chi-square test for association. This test checks whether there is a difference in the proportion distribution between the intervention and control groups; ^‡^Indicates a significant effect for p-value a ≤ 0.050 by the Chi-square test for proportion

Table 2 shows that, in Case 1, there was a statistically significant difference between the intervention and control groups regarding question 5 – “*Given Mrs. Catarina’s situation, would you report it to the 100 Hotline?“* - which assessed the attitudes of university students towards a situation of violence against the elderly.

**Table 2-:** Distribution of participants, intervention group and control group, according to the answer to each of the questions in Case 1 (n = 44). Marília, SP, Brazil, 2024

		**Group**				**Total**		*p* -value a*	***p* -value b** ^†^
		**Intervention (n=22)**		**Control (n=22)**					
		**N**	**%**	**N**	**%**	**N**	**%**		
Case 1 Q ^§^ 1	Yes	18	81.8%	17	77.3%	35	79.5%	<0.001 ^‡^	0.811
	No	1	4.5%	4	18.2%	5	11.4%		
	I can’t say for sure	3	13.6%	1	4.5%	4	9.1%		
Case 1 Q ^§^ 2	Yes	22	100.0%	22	100.0%	44	100.0%	NA ^||^	NA ^||^
Case 1 Q ^§^ 3	Yes	21	95.5%	21	95.5%	42	95.5%	<0.001 ^‡^	0.999
	Not enough knowledge	1	4.5%	1	4.5%	2	4.5%		
Case 1 Q ^§^ 4	Yes	20	90.9%	21	95.5%	41	93.2%	<0.001 ^‡^	0.554
	No definite position	2	9.1%	1	4.5%	3	6.8%		
Case 1 Q ^§^ 5	Yes	22	100.0%	12	54.5%	34	77.3%	<0.001 ^‡^	<0.001 ^¶^
	No	0	0.0%	3	13.6%	3	6.8%		
	No definite position	0	0.0%	7	31.8%	7	15.9%		
Case 1 Q ^§^ 6	Yes	21	95.5%	20	90.9%	41	93.2%	<0.001 ^‡^	0.307
	No	1	4.5%	0	0.0%	1	2.3%		
	I can’t say for sure	0	0.0%	2	9.1%	2	4.5%		

*p-value a = p-value calculated by the Chi-square test for proportion. This test checks whether there is a difference in the proportion distribution in the response categories as a whole; ^†^p-value b = p-value calculated by the Chi-square test for association. This test checks whether there is a difference in the proportion distribution between the intervention and control groups; ^‡^Indicates a significant effect for p-value a ≤ 0.050 by the Chi-square test for proportion; ^§^Q indicates the question in each Case; ^||^NA indicates that it was not possible to calculate the p-value, as everyone gave the same answer; ^¶^Indicates significant effect for p-value b ≤ 0.050 by the Chi-square test for association

In Cases 2, 3 and 5, there was no statistically significant difference between the intervention and control groups.

Table 3 shows that in Case 4, there was a statistically significant difference between the intervention and control groups regarding question 6 – “*Do you think that situations like this can be prevented?*” - which assessed the university students’ perceptions of how to prevent violence against the elderly in terms of their rights in society.

**Table 3-:** Distribution of participants, intervention group and control group, according to the answer to each of the questions in Case 4 (n = 44). Marília, SP, Brazil, 2024

		**Group**				**Total**		*p* -value a*	***p* -value b** ^†^
		**Intervention (n=22)**		**Control (n=22)**					
		N	**%**	**N**	**%**	**N**	**%**		
Case 4 Q ^§^ 1	Yes	17	77.3%	21	95.5%	38	86.4%	<0.001 ^‡^	0.078
	No	4	18.2%	1	4.5%	5	11.4%		
	I can’t say for sure	1	4.5%	0	0.0%	1	2.3%		
Case 4 Q ^§^ 2	Yes	3	13.6%	0	0.0%	3	6.8%	<0.001 ^‡^	0.106
	No	18	81.8%	20	90.9%	38	86.4%		
	Not enough knowledge	1	4.5%	2	9.1%	3	6.8%		
Case 4 Q ^§^ 3	Yes	17	77.3%	17	77.3%	34	77.3%	<0.001 ^‡^	0.675
	No	3	13.6%	1	4.5%	4	9.1%		
	Not enough knowledge	2	9.1%	4	18.2%	6	13.6%		
Case 4 Q ^§^ 4	Yes	1	4.5%	0	0.0%	1	2.3%	<0.001 ^‡^	0.209
	No	21	95.5%	21	95.5%	42	95.5%		
	No definite position	0	0.0%	1	4.5%	1	2.3%		
Case 4 Q ^§^ 5	Yes	17	77.3%	14	63.6%	31	70.5%	<0.001 ^‡^	0.463
	No	1	4.5%	3	13.6%	4	9.1%		
	No definite position	4	18.2%	5	22.7%	9	20.5%		
Case 4 Q ^§^ 6	Yes	1	4.5%	20	90.9%	21	47.7%	<0.001 ^‡^	<0.001 ^||^
	No	21	95.5%	0	0.0%	21	47.7%		
	I can’t say for sure	0	0.0%	2	9.1%	2	4.5%		

*p-value a = p-value calculated by the Chi-square test for proportion. This test checks whether there is a difference in the proportion distribution in the response categories as a whole; ^†^p-value b = p-value calculated by the Chi-square test for association. This test checks whether there is a difference in the proportion distribution between the intervention and control groups; ^‡^Indicates a significant effect for p-value a ≤ 0.050 by the Chi-square test for proportion; ^§^Q indicates the question in each Case; ^||^Indicates significant effect for p-value b ≤ 0.050 by the Chi-square test for association

When the questions from the five cases were grouped together, there was a significant difference between the intervention group for questions 5 and 6, where question 5 assessed attitudes and question 6 assessed university students’ perceptions (Table 4).

**Table 4-:** Distribution of participants, intervention and control group, according to response group (n = 44). Marília, SP, Brazil, 2024

**Variable**	**Group**	**Mean**	SD*	***p* -value** ^†^
**Q** ^‡^ **1% correct**	Intervention	67.273	29.3066	0.676
	Control	63.636	28.0383	
**Q** ^‡^ **2% correct**	Intervention	81.818	8.5280	0.060
	Control	77.273	7.0250	
**Q** ^‡^ **3% correct**	Intervention	93.636	11.3580	0.338
	Control	90.000	13.4519	
**Q** ^‡^ **4% correct**	Intervention	75.455	10.5683	0.504
	Control	72.727	15.7908	
**Q** ^‡^ **5% correct**	Intervention	57.273	9.3513	<0.001
	Control	41.818	15.0036	
**Q** ^‡^ **6% correct**	Intervention	79.091	4.2640	0.035 ^§^
	Control	89.091	21.1365	
**Question total correct answers %**	Intervention	75.758	6.6812	0.177 ^§^
	Control	72.424	9.2110	

*SD indicates Standard Deviation; ^†^p-value = p-value calculated by Student’s t-test; ^‡^Q indicates the question in each Case; ^§^Indicates significant difference between groups by Student’s t-test for p-value ≤ 0.050

## Discussion

This study examined the effect of an educational intervention on university students’ knowledge, perceptions, and attitudes toward violence against the elderly. The results showed significant differences between the intervention group and the control group in aspects related to attitudes and perceptions, especially on issues that require a more active stance, such as reporting cases of neglect and preventing situations of abuse.

In Case 1, which addressed family neglect, participants in the intervention group were more likely to report on the situation, suggesting that the intervention promoted practical and ethical awareness of the role of students and future professionals in dealing with abuse (p<0.001). This difference between the groups reinforces the effectiveness of educational strategies that integrate gamification and involve the simulation of practical scenarios, as they promote the development of protective attitudes in situations of neglect.

Family neglect of the elderly is a very subtle form of violence, often trivialized, “under-recognized“^(4)^ by society and especially by the aggressors themselves, who are not always aware that it is a form of violence.

Family neglect of older people is an issue of global relevance, characterized by a lack of essential care and support, which are particularly important at a stage of life when people may be most vulnerable^(17)^. In the family context, neglect is often motivated by factors such as caregiver stress and work overload^(18)^, combined with a lack of adequate support from public policies and health care institutions. Studies show that neglect, whether physical, emotional or financial, is one of the most common forms of elder abuse, particularly in countries where cultural traditions and social expectations assign the role of primary caregiver to family members^(19)^.

In developing countries such as Brazil, the phenomenon of family neglect is increasing due to factors such as the accelerated aging of the population and socioeconomic inequalities^(20)^. Neglect, which is often invisible and underreported, affects the quality of life of older people, depriving them of access to health care, adequate nutrition and emotional support. In addition, cultural and economic factors may influence the prevalence of abuse and neglect, with family members facing financial difficulties that reduce the availability of adequate support for their elderly members^(21)^.

Case 1 also emphasizes that reporting is essential to highlight and combat violence against older people. The low reporting rate is attributed to factors such as fear of reprisal, shame, and lack of knowledge about the channels available for reporting abuse. The World Health Organization (WHO) estimates that only a small fraction of cases of elder abuse are formally reported, suggesting that the problem is much larger than it appears^(1)^.

In Turkey, for example, a study of 161 family physicians found that the majority had limited knowledge of elder abuse and neglect, resulting in low rates of detection and reporting. This underreporting highlights the need for policies and support systems that facilitate and encourage reporting, in addition to training health professionals and caregivers to recognize signs of abuse and encouraging victims or family members to report^(22)^.

In addition, cultural barriers can influence the decision to report, as seen in India and in some cultural groups in the United States, where the value placed on family relationships or fear of stigma make it difficult to initiate formal proceedings against perpetrators^(23)^. Strengthening awareness campaigns and establishing safe and confidential helplines are necessary measures to overcome these barriers and ensure that older people are guaranteed their rights and protected from abuse^(24)^.

These initiatives aim to make the act of reporting an accessible and safe step for older people and their families, as well as to raise awareness in society about the seriousness and consequences of violence against older people, thus helping to reduce its invisibility and build a more effective protection network.

In Case 4, concerning disrespect for the rights of the elderly, there was a significant difference between the groups, with students in the intervention group showing a greater perception of the need for prevention and respect for the rights of the elderly in public spaces (p<0.001). This result suggests that the intervention was not only effective in transferring knowledge, but also in promoting an ethical attitude towards everyday situations, recognizing the citizenship of the elderly.

The rights of older people have been discussed and addressed by various organizations, including WHO and the United Nations (UN)^(1)^. Despite progress, the effectiveness of policies varies widely from country to country. Some regions, such as Europe, have more robust legislation and enforcement mechanisms to protect older people from neglect and other abuse. On the other hand, in many developing countries, a lack of resources and specific policies prevents older people’s rights from being effectively guaranteed and respected. The COVID-19 pandemic has also exposed critical vulnerabilities, particularly in long-term care facilities, where neglect has proved fatal in many cases due to the lack of adequate safety and care protocols^(1-4)^.

In Brazil, public policies for the elderly have evolved significantly, reflecting a response to the growing needs of this age group. The promulgation of the National Policy for the Elderly in 1994 was a first milestone, guaranteeing basic social rights for the elderly and establishing guidelines for their integration and appreciation in society. In 1999, the National Health Policy for the Elderly reinforced this commitment by defining specific responsibilities for promoting the health and well-being of this population, with a view to active and healthy ageing^(25)^.

The creation of the Senior Citizens’ Statute^(5)^ further consolidated these rights, and it is widely recognized as one of the country’s greatest social achievements. The Statute expanded the responsibilities of both the State and society in protecting and meeting the needs of older people, guaranteeing rights in areas such as health care, social welfare and protection from violence and neglect^(5)^.

Educational interventions play a critical role in preventing violence against older people and are recognized worldwide as an effective tool in addressing the problem. These actions include training of health professionals, public awareness campaigns, and the inclusion of educational content on respect for the elderly in school curricula. In many countries, such as India, these strategies have been implemented to promote intergenerational empathy and awareness of the signs of abuse^(23)^.

Education also aims to prepare caregivers and family members to deal with ageing and the specific needs of older people, to avoid situations of abuse and neglect that may arise from a lack of knowledge or preparation. In addition, the formation of multidisciplinary teams and the introduction of family support programs are effective forms of prevention^(21-22)^.

In addition, WHO emphasizes the importance of educational measures in the context of the Decade of Healthy Ageing 2020-2030, which promotes the training of health care professionals to identify and respond to cases of mistreatment using a human rights-based approach and to ensure that older people have access to safe and respectful environments^(26-27)^.

These educational initiatives, combined with appropriate public policies, form a necessary global approach to protecting and valuing the rights of older people and contribute to building more inclusive societies free from violence against the elderly.

The effectiveness of the educational intervention in changing perceptions and attitudes observed in the intervention groups is in line with the literature highlighting the importance of active teaching methods in health care and the humanities to engage students in learning processes that go beyond theoretical knowledge and promote an intersectoral understanding of the human rights of older persons^(28)^.

Active learning methods are innovative teaching modalities in the context of health education as they seek to articulate theory and practice through meaningful learning and to provide the necessary changes to implement preventive care to reduce violence against the elderly, thus strengthening the construction of knowledge in an interdisciplinary manner^(29)^.

Although the results highlight the value of interventions using gamification in training professional’s sensitive to the realities of the elderly, they are specific to the context studied and should not be generalized to other situations or settings.

The results obtained contribute to highlight the need to expand the use of innovative and interactive methodologies, such as gamification, in university curricula, as demonstrated in a study on the benefits of gamification in medical education^(30)^. These approaches seem to be effective in promoting attitudes of protection and respect for the elderly, acting as a tool for social change and violence prevention.

This study has some limitations that should be considered when interpreting the results. First, since this is a non-randomized clinical trial, the lack of randomization may have introduced selection biases that affect the representativeness and generalizability of the results. Although the groups were paired to minimize these differences, this method does not ensure the complete elimination of confounding variables.

Although the questionnaire was validated by referees, responses that were more in line with social expectations may have been influenced by the nature of the instrument and the participants’ awareness of the aim of the intervention. In addition, another influence may be related to the individual characteristics of the participants, such as their major and previous experience with older people. Finally, the sample size was limited.

This study contributes to the advancement of scientific knowledge by demonstrating that the gamified intervention can be an effective strategy for changing university students’ attitudes and perceptions about violence against the elderly, promoting greater awareness and a greater propensity to report cases of neglect. However, the lack of significant improvement in knowledge suggests the need for other complementary educational interventions. In addition, the study points to the importance of future research, especially longitudinal studies, to assess the sustainability of these changes over time.

## Conclusion

This study highlighted the importance of gamified educational interventions in developing protective attitudes and increasing awareness of violence against older adults among university students. Through educational activities based on gamification, it was possible to engage participants in an active process that promoted more practical and applied learning. Although it did not lead to significant improvements in theoretical knowledge, it did increase participants’ awareness of the seriousness of violence against older adults. The results suggest that active methods, such as gamification, are effective in stimulating behavioral change and promoting empathy and respect for the rights of older persons.

It is concluded that in the context of this study, interactive educational practices that are sensitive to the needs of older adults can help train professionals to be more aware of elder abuse. However, these findings are specific to the setting studied and should not be generalized to other contexts.
