# Erratum: The effect of an educational intervention for university students on violence against the elderly using gamification: a non-randomized clinical trial

**DOI:** 10.1590/1518-8345.0000.4741

**Published:** 2025-10-27

Regarding the article “The effect of an educational intervention for university students on violence against the elderly using gamification: a non-randomized clinical trial”, with DOI number: 10.1590/1518-8345.7874.4662, published in the Rev. Latino-Am. Enfermagem, 2025;33:e4662:

In the Results section, in the 4th paragraph, page 6:

Where was written:

“In Cases 2, 3 and 5”

Now read:

“In Cases 2, 3, 4 and 5”

In the Results section, in the 5th paragraph, page 6:

Where was written:

“Table 3 shows that in Case 4, there was a statistically significant difference between the intervention and control groups”

Now read:

“Table 3 shows that in Case 4, there was no statistically significant difference between the intervention and control groups”

In the Results section, in Table 3, page 6:

Where was written:

**Table:** 

		**Group**				**Total**		*p* -value a*	***p* -value b** ^†^
		**Intervention (n=22)**		**Control (n=22)**					
		N	**%**	**N**	**%**	**N**	**%**		
Case 4 Q ^§^ 6	Yes	1	4.5%	20	90.9%	21	47.7%	<0.001 ^‡^	<0.001 ^||^
	No	21	95.5%	0	0.0%	21	47.7%		
	I can’t say for sure	0	0.0%	2	9.1%	2	4.5%		

Now read:

**Table:** 

		**Group**				**Total**		*p* -value a*	***p* -value b** ^†^
		**Intervention (n=22)**		**Control (n=22)**					
		N	**%**	**N**	**%**	**N**	**%**		
Case 4 Q ^§^ 6	Yes	21	95.5%	20	90.9%	41	93.2%	<0.001 ^‡^	<0.220
	No	1	4.5%	0	0.0%	1	2.3%		
	I can’t say for sure	0	0.0%	2	9.1%	2	4.5%		

In the Results section, in the 6th paragraph, page 6:

Where was written:

“When the questions from the five cases were grouped together, there was a significant difference between the intervention group for questions 5 and 6, where question 5 assessed attitudes and question 6 assessed university students’ perceptions (Table 4).”

Now read:

“When the questions from the five cases were grouped together, there was a significant difference between the intervention group for questions 5, which assessed the attitudes of university students (Table 4).”

In the Results section, in Table 4, page 7:

Where was written:

**Table:** 

**Variable**	**Group**	**Mean**	SD*	***p* -value** ^†^
**Q** ^‡^ **6% correct**	Intervention	79.091	4.2640	0.035 ^§^
	Control	89.091	21.1365	
**Question total correct answers %**	Intervention	75.758	6.6812	0.177 ^§^
	Control	72.424	9.2110	

Now read:

**Table:** 

**Variable**	**Group**	**Mean**	SD*	***p* -value** ^†^
**Q** ^‡^ **6% correct**	Intervention	97.3	12.79	0.128
	Control	89.1	21.14	
**Question total correct answers %**	Intervention	78.8	7.38	0.015 ^§^
	Control	72.4	9.18	

In the Conclusion section, in the 1st paragraph, page 9:

Where was written:

“This study highlighted the importance of gamified educational interventions in developing protective attitudes and increasing awareness of violence against older adults among university students.”

Now read:

“This study highlighted the importance of gamified educational interventions in developing protective attitudes towards elder abuse among university students.”

In the Highlights section on page 1, the following information was removed: “**(2)** Increased awareness of the rights of older people in public spaces.”.

In the Summary section, under Results, on page 1, the following information was removed: “and perceptions”.

In the Results section, in the 5th paragraph on page 6, the following information was removed: “regarding question 6 –”*Do you think that situations like this can be prevented?*” - which assessed the university students’ perceptions of how to prevent violence against the elderly in terms of their rights in society.”.

In the Discussion section, in the 1st paragraph (line 6) on page 7, the following information was removed: “and perceptions”.

In the Discussion section, page 8, the 10th paragraph was completely removed.

In the Discussion section, in the last paragraph (line 4) on page 9, the following information was removed: “and perceptions”.

