# Supplementary material for: The effect of an educational intervention for university students on violence against the elderly using gamification: a non-randomized clinical trial
Source: Rev Lat Am Enfermagem. 2025 Aug 18;33:e4662. doi: 10.1590/1518-8345.7874.4662 (PMC12364459; doi:10.1590/1518-8345.7874.4662)
Supplement: Translation

# Translation: Efeito de uma intervenção educativa para universitários sobre violência contra pessoas idosas por meio da gamificação: ensaio clínico não randomizado*

**DOI:** 10.1590/1518-8345.7874.4663

**Authors:** Juliana Ribeiro da Silva Vernasque, Miriam Fernanda Sanches Alarcon, Daiana Bonfim, Paula Sales Rodrigues, Eduardo Federighi Baisi Chagas, Maria José Sanches Marin

**Affiliations:** 1 Faculdade de Medicina de Marília, Educação em Ciências da Saúde, Marília, SP, Brasil.; 2 Universidade Estadual Paulista, Departamento de Enfermagem, Botucatu, SP, Brasil.; 3 Bolsista da Coordenação de Aperfeiçoamento de Pessoal de Nível Superior (CAPES), Brasil.; 4 Universidade Estadual do Norte do Paraná, Departamento de Enfermagem, Bandeirantes, PR, Brasil.; 5 Faculdade Israelita de Ciências da Saúde Albert Einstein, Centro de Estudos, Pesquisa e Prática APS e Redes, São Paulo, SP, Brasil.; 6 Faculdade de Medicina de Marília, Estatística, Marília, SP, Brasil.; 7 Faculdade de Medicina de Marília, Enfermagem em Saúde Coletiva, Marília, SP, Brasil.

**Keywords:** Idoso, Violência, Universidades, Abuso de Idosos, Atitude, Conhecimento

## Abstract

avaliar o impacto de uma intervenção educativa gamificada sobre violência contra a pessoa idosa, no conhecimento, percepção e atitude de universitários.

ensaio clínico não randomizado com 44 universitários de cursos das áreas das ciências da saúde, humanas e exatas, sendo 22 do grupo de intervenção e 22 do grupo controle. O grupo de intervenção participou de atividades híbridas sobre gamificação aplicada ao tema da violência contra idosos. A avaliação foi feita com *cases* temáticos validados por especialistas, com análise estatística usando testes do Qui-quadrado e t de Student.

a intervenção promoveu diferenças significativas entre os grupos em atitudes e percepções sobre violência contra pessoas idosas. Estudantes do grupo de intervenção mostraram maior propensão a denunciar casos de negligência e maior conscientização sobre a prevenção de abusos em espaços públicos.

a intervenção gamificada demonstrou efetividade em estimular atitudes protetoras e percepção ética diante da violência contra pessoas idosas, porém não houve associação significativa com a variável conhecimento. Depreende-se a necessidade de estudos mais abrangentes que possam contribuir com evidências complementares aos resultados do presente estudo.

## Destaques

**(1)** Universitários relatam maior propensão a denunciar negligência familiar. **(2)** Aumento da percepção sobre os direitos das pessoas idosas em espaços públicos. **(3)** Intervenção favorece conscientização prática sobre abuso de pessoas idosas. **(4)** Abordagens inovadoras são eficazes na formação de profissionais mais empáticos. **(5)** Intervenção gamificada promove atitudes protetoras em relação às pessoas idosas.

## Introdução

A violência contra a pessoa idosa, caracterizada por ações ou omissões que causam danos ou sofrimento a essa população, é um problema mundialmente reconhecido^(1)^. Estima-se que, para cada caso registrado, outros cinco não são notificados, evidenciando a subnotificação e a invisibilidade do problema^(2)^.

A violência contra a pessoa idosa assume várias formas − abusos físicos, psicológicos, sexuais, financeiros e negligência − e cada uma delas causa impactos severos e exige abordagens intersetoriais para a sua identificação e combate. Notavelmente, a maioria dos agressores são familiares, o que dificulta a denúncia e a intervenção^(3)^. O reconhecimento da violência contra a pessoa idosa ainda ocorre de forma tardia e inadequada entre as diferentes áreas do conhecimento, sendo considerado um problema “subfinanciado, subexaminado e sub-reconhecido”^(4)^.

Identificar e denunciar situações em que a pessoa idosa esteja sofrendo violência é um processo complexo e cauteloso. Além disso, há desconhecimento dos diferentes setores da sociedade e da própria pessoa idosa sobre os direitos e significado da violência^(5)^.

Estudos apontam que ações educativas podem ser efetivas na prevenção do abuso contra as pessoas idosas. Um estudo transversal realizado nos Estados Unidos da América (EUA), com 2.150 idosos coreano-americanos, analisou a prevalência e os fatores associados aos maus-tratos físicos, emocionais e financeiros, concluindo que são necessárias estratégias preventivas e intervenções educativas contextualizadas^(6)^. Em Israel, um estudo com 145 ortopedistas evidenciou que conhecimento e atitudes mais positivas estavam associados a um aumento na identificação e no relato de casos, ressaltando a importância do treinamento na detecção e no manejo do abuso contra as pessoas idosas desde o início da carreira^(7)^. Além disso, foi demonstrado que treinamentos *online* em uma universidade dos Estados Unidos da América (EUA)^(8)^ melhoraram significativamente o conhecimento de estudantes sobre a interação adequada com idosos vítimas de abusos. Resultados semelhantes, foram encontrados entre enfermeiros iranianos^(9)^, uma vez que níveis mais elevados de educação se associaram a melhores práticas assistenciais, embora barreiras como carga de trabalho e falta de treinamento ainda dificultem a aplicação do conhecimento.

Nesse contexto, entende-se que ações educativas interdisciplinares que promovam uma aprendizagem significativa são essenciais para estimular não apenas o conhecimento, mas também percepções e atitudes protetoras em relação à população idosa. Ao envolver os estudantes em uma formação gamificada que integre valores e competências práticas, espera-se fomentar uma compreensão mais profunda e atitudes de respeito e proteção, necessárias para enfrentar essa problemática complexa.

A gamificação consiste no uso de elementos e técnicas de *design* de *games* na Educação, na Saúde e no mundo corporativo. No contexto educacional, a gamificação, especialmente quando combinada com aprendizado colaborativo, contribui para o desenvolvimento de competências transversais, como criatividade, resolução de problemas e trabalho em equipe, além de melhorar o desempenho acadêmico e a motivação dos estudantes^(10)^.

Dessa maneira, a pergunta de pesquisa elaborada é: jovens universitários capacitados para o reconhecimento de violência contra a pessoa idosa por meio da gamificação melhoram o conhecimento, a atitude e a percepção, resultando em maior responsabilidade do que em jovens universitários não treinados?

O objetivo deste estudo é avaliar o impacto de uma intervenção educativa gamificada sobre violência contra a pessoa idosa, no conhecimento, percepção e atitude de universitários.

## Método

### Delineamento do estudo

Trata-se de um ensaio clínico não randomizado. Para manter o rigor metodológico, foi utilizado o protocolo *Standards for QUality Improvement Reporting Excellence in Education* (SQUIRE 2.0) como ferramenta de apoio para o desenvolvimento da pesquisa^(11)^.

O desenho não randomizado foi escolhido em virtude da natureza do estudo e das características do contexto de aplicação^(12)^. Dada a dificuldade em randomizar a alocação dos participantes em grupos de intervenção, o estudo optou por envolver os universitários disponíveis e dispostos a participar, sem a necessidade de uma divisão aleatória entre grupos. Essa abordagem permitiu a aplicação de uma intervenção educativa em um ambiente real, proporcionando uma análise mais flexível e prática dos impactos sobre o conhecimento, percepção e atitude dos participantes.

### Local da pesquisa

O local de realização da pesquisa foi um município de médio porte, do Centro-Oeste do Estado de São Paulo (Brasil), com uma população estimada em 235.234 habitantes^(13)^. O referido município conta com um grande complexo de instituições de ensino superior (IES), tanto de caráter público como privado, as quais oferecem cursos em diferentes áreas do conhecimento. Para o presente estudo, foram incluídas quatro IES, sendo duas públicas e duas privadas, com vistas a contar com universitários dos cursos das áreas das ciências da saúde, exatas e humanas.

### Período

O estudo foi realizado no período de dezembro de 2022 a abril de 2024.

### População

Participaram deste estudo os universitários provenientes dos cursos da saúde, humanas e exatas, selecionados de forma não aleatória, dado o contexto e a natureza da intervenção educativa. Foram constituídos dois grupos: o primeiro (grupo de intervenção), composto por jovens universitários que participaram da intervenção educativa; e o segundo (grupo controle), composto por jovens universitários que não participaram da intervenção educativa. Durante o período do estudo, esses participantes mantiveram suas atividades acadêmicas regulares, sem envolvimento em atividades relacionadas ao tema da intervenção ou qualquer modificação específica em sua rotina acadêmica.

### Critérios de seleção

O grupo de intervenção foi recrutado em dezembro de 2022, para participar durante um ano, até dezembro de 2023. Inicialmente, para a seleção dos participantes (grupo de intervenção), foram reunidos 40 jovens universitários: estudantes dos cursos de Enfermagem, Medicina, Direito, Terapia Ocupacional, Fisioterapia, Engenharia Civil, Psicologia e Pedagogia. A seleção ocorreu conforme o interesse demonstrado pelos estudantes em participar da intervenção educativa. Do processo completo, participaram 22 universitários, contemplando os seguintes cursos: Enfermagem, Medicina, Direito, Terapia Ocupacional e Fisioterapia.

A seleção dos integrantes do grupo controle ocorreu por conveniência, sendo recrutado em março de 2024, e não foi realizada nenhuma intervenção. Para a composição dos grupos, foram realizados convites com a colaboração de docentes e coordenadores dos cursos das instituições participantes. O delineamento da intervenção foi controlado; os grupos foram constituídos por pareamento, garantindo uma composição rigorosa e aberta^(12)^.

Os critérios de inclusão na pesquisa foram os seguintes: ser estudante universitário; estar matriculado em uma das quatro instituições de ensino superior participantes; e, no caso do grupo de intervenção, ter disponibilidade para participar, ao longo de um ano, das atividades educativas. Foram excluídos do estudo: os universitários que não pertenciam aos cursos das áreas de ciências da saúde, humanas e exatas; estudantes que não possuíam acesso a dispositivos digitais ou à internet e indivíduos que não preencheram o instrumento de coleta de dados.

### Intervenção

A intervenção educativa ocorreu ao longo de um ano, com quatro encontros presenciais, intercalados com encontros *online,* totalizando uma carga horária total de 50 horas. O processo educativo utilizou-se da gamificação, por meio de conferências, aulas expositivas dialogadas e oficinas de trabalho, nas quais os estudantes foram estimulados a refletir sobre a violência contra a pessoa idosa, a partir das próprias vivências, bem como sobre a possiblidades de desenvolvimento de *games* com vistas a promover a saúde da pessoa idosa e prevenir a violência. As atividades foram apoiadas por docentes e pesquisadores sobre o tema “Violência Contra a Pessoa Idosa” e por um especialista em *games*.

Entende-se que a utilização de tecnologias da informação nos processos educativos, em todos os campos do saber, pode potencializar a aprendizagem significativa e contribuir para a construção de saberes práticos e mudança de atitudes.

No primeiro encontro presencial, houve uma apresentação sobre o tema “Violência Contra a Pessoa Idosa”, abordando conceitos importantes, aspectos epidemiológicos e resultados de estudos realizados no próprio município, que evidenciam a problemática enfrentada por essa parcela da população^(14)^.

Na sequência, foi realizada uma exposição dialógica sobre os princípios e etapas da gamificação, destacando a interseção entre as duas temáticas: gamificação e violência contra a pessoa idosa.

Na dinâmica dos trabalhos, os estudantes foram divididos em grupos com base em afinidades para criar propostas de *games* com a temática violência contra a pessoa idosa. Em cada nova reunião, os grupos apresentavam o progresso em seus trabalhos, seguido de um espaço de discussão para que os demais participantes pudessem analisar e avaliar os projetos em desenvolvimento, oferecendo sugestões para o aprimoramento das propostas. Assim, as propostas de cada grupo foram construídas e refinadas dentro dos pequenos grupos e com a contribuição de todos os participantes.

Ao término, foram elaborados seis projetos com gamificação, permitindo que os estudantes envolvidos pudessem se engajar permanentemente ao longo do período, tanto em relação à temática como na construção efetiva de um projeto que pudesse ter aplicação prática na sociedade.

### Variáveis do estudo

Para caracterização da amostra, foram coletados dados sobre a idade, sexo e curso de graduação. Para avaliar a efetividade da intervenção, foi considerado como desfecho primário o percentual de acertos para os casos propostos. Foram elaborados cinco *cases,* tendo cada um deles a intencionalidade de abordar uma forma específica de violência contra a pessoa idosa, permitindo uma análise ampla dos diferentes tipos de abuso.

Para validação dos *cases* pelos juízes, foi adotada uma escala Likert, permitindo que os avaliadores expressassem o grau de concordância com a adequação de cada pergunta à avaliação pretendida.

Esse processo buscou garantir que as perguntas capturassem com precisão as percepções, o conhecimento técnico e as atitudes dos estudantes diante de diferentes situações de violência enfrentadas por idosos, em diversos contextos. Além disso, disponibilizou-se um espaço para sugestões e opiniões sobre determinado conteúdo quando o avaliador julgasse necessário. A validação do conteúdo dos *cases* foi realizada com a participação de 11 juízes convidados, com expertise, ou na área de geriatria e gerontologia, ou em processos de avaliação de aprendizagem, e eles assinaram o Termo de Consentimento Livre e Esclarecido.

Em relação às características dos juízes: nove do gênero feminino e dois do gênero masculino; a média de idade: 53 anos; a formação profissional: três com graduação em Enfermagem, quatro em Medicina, dois em Psicologia, um em Ciências Farmacêuticas e um em Assistência Social. Quanto ao tempo de formação, a média em anos foi de 29,54 e a média do tempo de atuação na atividade atual foi de 26,54 anos. A validação aconteceu em dois momentos distintos, considerando que os juízes apresentaram sugestões pertinentes que foram acrescentadas ao instrumento de avaliação.

Utilizou-se o Coeficiente de Validade de Conteúdo (CVC)^(15)^, para analisar o nível de concordância entre os juízes. O CVC é um indicador recomendado para calcular o nível de consenso, mediante o cálculo da média do valor atribuído pelos juízes em cada item do instrumento, expresso em termos percentuais. Foram calculados o CVC individual para cada questão e a média de todos para o CVC global, sendo considerados válidos os itens com percentuais iguais ou superiores a 80%^(16)^. Para o instrumento com os cinco *cases* e as seis questões em cada *case*, foi calculado o CVC, sendo que, nos dois momentos, este foi de 0,94.

Os *cases* validados foram inseridos em um formulário do *Google Forms* e, posteriormente, enviados via *WhatsApp* para os participantes, tanto para o grupo de intervenção como para o grupo controle. O *Case 1* foca na negligência familiar, examinando se o estudante consegue identificar sinais de descuido e omissão no cuidado com idosos. O *Case 2* trata da violência financeira, avaliando a percepção dos alunos sobre a exploração econômica e o controle indevido de bens do idoso. No *Case 3*, a ênfase é na violência física em uma instituição de longa permanência, buscando avaliar o reconhecimento dos sinais de agressão física. O *Case 4* aborda o desrespeito aos direitos da pessoa idosa no transporte público, incentivando os estudantes a refletirem sobre atitudes de cidadania e respeito. Por fim, o *Case 5* explora a violência sexual, focando em identificar o abuso e as consequências do relacionamento abusivo.

Para cada *case*, seguiram-se seis perguntas com o intuito de avaliar conhecimentos, percepções e atitudes dos participantes. As perguntas um e seis foram direcionadas para a avaliação da percepção dos estudantes em relação à violência contra a pessoa idosa na sociedade; as perguntas dois e três, para aferir o conhecimento; e as perguntas quatro e cinco, para avaliar atitudes. Cada questão foi respondida com opções que variavam entre “sim”, “não”, “não posso afirmar com certeza”, “sem conhecimento suficiente” e “sem posicionamento definido”. No entanto, para o cálculo do percentual de acerto foi considerado somente o somatório das respostas corretas.

### Análise dos dados

As variáveis qualitativas estão descritas pela distribuição de frequência absoluta (N) e relativa (%). Para analisar a diferença de distribuição de frequência, utilizou-se o teste do Qui-quadrado para proporção. Para analisar a relação entre variáveis qualitativas e o grupo, utilizou-se o teste de associação do Qui-quadrado. Foi utilizado o teste t de Student para amostras independentes para comparação de médias após a verificação de homogeneidade das variâncias pelo teste de Levene. O nível de significância adotado foi de 5%, e os dados foram analisados no *software* SPSS (versão 27.0).

### Aspectos éticos

A pesquisa recebeu aprovação do Comitê de Ética em Pesquisa (CEP), sob o Parecer n.º 5.144.186, e todos os participantes assinaram o Termo de Consentimento Livre e Esclarecido.

## Resultados

Participaram 22 estudantes do grupo de intervenção e 22 estudantes do grupo controle. Em relação à idade, a média do grupo de intervenção foi de 23 anos, com 2,11 de desvio-padrão; e a média do grupo controle foi de 24,04, com 2,11 de desvio-padrão.

Verifica-se a homogeneidade dos grupos de intervenção e controle, no que se refere ao gênero e cursos de graduação (Tabela 1).

**Tabela 1-:** Distribuição dos participantes, grupo de intervenção e grupo controle, segundo o sexo e curso de graduação (n = 44). Marília, SP, Brasil, 2024

		**Grupo**				**Total**		*p* -valor a*	***p* -valor b** ^†^
		**Intervenção (n=22)**		**Controle (n=22)**					
		**N**	**%**	**N**	**%**	**N**	**%**		
Sexo	Masculino	5	22,7%	5	22,7%	10	22,7%	<0,001 ^‡^	0,999
	Feminino	17	77,3%	17	77,3%	34	77,3%		
Curso	Medicina	10	45,5%	12	54,5%	22	50,0%	<0,001 ^‡^	0,904
	Enfermagem	8	36,4%	5	22,7%	13	29,5%		
	Terapia Ocupacional	1	4,5%	1	4,5%	2	4,5%		
	Direito	2	9,1%	2	9,1%	4	9,1%		
	Fisioterapia	1	4,5%	2	9,1%	3	6,8%		

*p-valor a = p-valor calculado pelo teste do Qui-quadrado para proporção. Esse teste verifica se existe diferença na distribuição de proporção nas categorias de respostas no total; ^†^p-valor b = p-valor calculado pelo teste do Qui-quadrado para associação. Esse teste verifica se existe diferença na distribuição de proporção entre o grupo de intervenção e controle; ^‡^Indica efeito significativo para p-valor a ≤ 0,050 pelo teste do Qui-quadrado para proporção

Na Tabela 2, observa-se que no *Case* 1 houve diferença significativamente estatística entre os grupos de intervenção e controle, no que se refere à questão 5 − *“Diante da situação da Sra. Catarina, você faria uma denúncia no disque 100?”* −, que avaliou a atitude do universitário em relação a uma situação de violência contra a pessoa idosa.

**Tabela 2-:** Distribuição dos participantes, grupo de intervenção e grupo controle, conforme resposta para cada uma das perguntas do *Case*1 (n = 44). Marília, SP, Brasil, 2024

		**Grupo**				**Total**		*p* -valor a*	***p* -valor b** ^†^
		**Intervenção (n=22)**		**Controle (n=22)**					
		**N**	**%**	**N**	**%**	**N**	**%**		
*Case* 1 Q ^§^ 1	Sim	18	81,8%	17	77,3%	35	79,5%	<0,001 ^‡^	0,811
	Não	1	4,5%	4	18,2%	5	11,4%		
	Não posso afirmar com certeza	3	13,6%	1	4,5%	4	9,1%		
*Case* 1 Q ^§^ 2	Sim	22	100,0%	22	100,0%	44	100,0%	NA ^||^	NA ^||^
*Case* 1 Q ^§^ 3	Sim	21	95,5%	21	95,5%	42	95,5%	<0,001 ^‡^	0,999
	Sem conhecimento suficiente	1	4,5%	1	4,5%	2	4,5%		
*Case* 1 Q ^§^ 4	Sim	20	90,9%	21	95,5%	41	93,2%	<0,001 ^‡^	0,554
	Sem posicionamento definido	2	9,1%	1	4,5%	3	6,8%		
*Case* 1 Q ^§^ 5	Sim	22	100,0%	12	54,5%	34	77,3%	<0,001 ^‡^	<0,001 ^¶^
	Não	0	0,0%	3	13,6%	3	6,8%		
	Sem posicionamento definido	0	0,0%	7	31,8%	7	15,9%		
*Case* 1 Q ^§^ 6	Sim	21	95,5%	20	90,9%	41	93,2%	<0,001 ^‡^	0,307
	Não	1	4,5%	0	0,0%	1	2,3%		
	Não posso afirmar com certeza	0	0,0%	2	9,1%	2	4,5%		

*p-valor a = p-valor calculado pelo teste do Qui-quadrado para proporção. Esse teste verifica se existe diferença na distribuição de proporção nas categorias de respostas no total; ^†^p-valor b= p-valor calculado pelo teste do Qui-quadrado para associação. Esse teste verifica se existe diferença na distribuição de proporção entre o grupo de intervenção e controle; ^‡^Indica efeito significativo para p-valor a ≤ 0,050 pelo teste do Qui-quadrado para proporção; ^§^Q = Indica a questão de cada *Case*; ^||^NA = Indica que não foi possível calcular o p-valor, pois todos apresentaram a mesma resposta; ^¶^Indica efeito significativo para p-valor b ≤ 0,050 pelo teste de associação do Qui-quadrado

Nos *Cases* 2, 3, e 5, não houve diferença significativamente estatística entre os grupos de intervenção e controle.

Na Tabela 3, observa-se que, no *Case* 4, houve diferença significativamente estatística entre os grupos de intervenção e controle, no que se refere à questão 6 − *“Você considera que situações como esta podem ser prevenidas?”* −, que avaliou a percepção do universitário em relação à prevenção de uma situação de violência contra a pessoa idosa, no que tange aos seus direitos na sociedade.

**Tabela 3-:** Distribuição dos participantes, grupo de intervenção e grupo controle, conforme resposta para cada uma das perguntas do *Case*4 (n = 44). Marília, SP, Brasil, 2024

		**Grupo**				**Total**		*p* -valor a*	***p* -valor b** ^†^
		**Intervenção (n=22)**		**Controle (n=22)**					
		N	**%**	**N**	**%**	**N**	**%**		
*Case* 4 Q ^§^ 1	Sim	17	77,3%	21	95,5%	38	86,4%	<0,001 ^‡^	0,078
	Não	4	18,2%	1	4,5%	5	11,4%		
	Não posso afirmar com certeza	1	4,5%	0	0,0%	1	2,3%		
*Case* 4 Q ^§^ 2	Sim	3	13,6%	0	0,0%	3	6,8%	<0,001 ^‡^	0,106
	Não	18	81,8%	20	90,9%	38	86,4%		
	Sem conhecimento suficiente	1	4,5%	2	9,1%	3	6,8%		
*Case* 4 Q ^§^ 3	Sim	17	77,3%	17	77,3%	34	77,3%	<0,001 ^‡^	0,675
	Não	3	13,6%	1	4,5%	4	9,1%		
	Sem conhecimento suficiente	2	9,1%	4	18,2%	6	13,6%		
*Case* 4 Q ^§^ 4	Sim	1	4,5%	0	0,0%	1	2,3%	<0,001 ^‡^	0,209
	Não	21	95,5%	21	95,5%	42	95,5%		
	Sem posicionamento definido	0	0,0%	1	4,5%	1	2,3%		
*Case* 4 Q ^§^ 5	Sim	17	77,3%	14	63,6%	31	70,5%	<0,001 ^‡^	0,463
	Não	1	4,5%	3	13,6%	4	9,1%		
	Sem posicionamento definido	4	18,2%	5	22,7%	9	20,5%		
*Case* 4 Q ^§^ 6	Sim	1	4,5%	20	90,9%	21	47,7%	<0,001 ^‡^	<0,001 ^||^
	Não	21	95,5%	0	0,0%	21	47,7%		
	Não posso afirmar com certeza	0	0,0%	2	9,1%	2	4,5%		

*p-valor a = p-valor calculado pelo teste do Qui-quadrado para proporção. Esse teste verifica se existe diferença na distribuição de proporção nas categorias de resposta no total; ^†^p-valor b = p-valor calculado pelo teste do Qui-quadrado para associação. Esse teste verifica se existe diferença na distribuição de proporção entre o grupo de intervenção e controle; ^‡^Indica efeito significativo para p-valor a ≤ 0,050 pelo teste do Qui-quadrado para proporção; ^§^Q = Indica a questão de cada *Case*; ^||^Indica efeito significativo para p-valor b ≤ 0,050 pelo teste de associação do Qui-quadrado

Quando agrupadas as questões dos cinco *cases*, verifica-se que houve diferença significativa entre o grupo de intervenção para as questões 5 e 6, sendo que a questão 5 avaliou as atitudes, e a questão 6, as percepções dos universitários (Tabela 4).

**Tabela 4-:** Distribuição dos participantes, grupo de intervenção e controle, conforme o grupo de respostas (n = 44). Marília, SP, Brasil, 2024

**Variável**	**Grupo**	**Média**	DP*	***p* -valor** ^†^
Q ^‡^ 1% acertos	Intervenção	67,273	29,3066	0,676
	Controle	63,636	28,0383	
Q ^‡^ 2% acertos	Intervenção	81,818	8,5280	0,060
	Controle	77,273	7,0250	
Q ^‡^ 3% acertos	Intervenção	93,636	11,3580	0,338
	Controle	90,000	13,4519	
Q ^‡^ 4% acertos	Intervenção	75,455	10,5683	0,504
	Controle	72,727	15,7908	
Q ^‡^ 5% acertos	Intervenção	57,273	9,3513	<0.001
	Controle	41,818	15,0036	
Q ^‡^ 6% acertos	Intervenção	79,091	4,2640	0.035 ^§^
	Controle	89,091	21,1365	
Questão total acertos %	Intervenção	75,758	6,6812	0,177 ^§^
	Controle	72,424	9,2110	

*DP = Desvio-Padrão; ^†^p-valor = p-valor calculado pelo teste t de Student; ^‡^Q = Indica a questão de cada *Case*; ^§^Indica diferença significativa entre os grupos pelo teste t de Student para p-valor ≤ 0,050

## Discussão

A presente pesquisa investigou o efeito de uma intervenção educativa sobre o conhecimento, percepção e atitudes de universitários em relação à violência contra a pessoa idosa. Os resultados mostraram diferenças significativas entre o grupo de intervenção e o grupo controle em aspectos relacionados a atitudes e percepções, especialmente nas questões que exigiam posicionamentos mais ativos, como a denúncia de casos de negligência e a prevenção de situações de abuso.

No *Case* 1, que abordou a negligência familiar, observou-se que os participantes do grupo de intervenção apresentaram maior propensão a denunciar a situação, sugerindo que a intervenção promoveu uma conscientização prática e ética sobre o papel do estudante e futuro profissional, no enfrentamento de abusos (p<0,001). Essa diferença entre os grupos reforça a efetividade das estratégias educativas que integram gamificação e envolvem simulação de cenários práticos, pois potencializam o desenvolvimento de atitudes protetoras em situações de negligência.

A negligência familiar em relação às pessoas idosas é um tipo de violência muito sutil, muitas vezes banalizado, “sub-reconhecido”^(4)^ por parte da sociedade e, principalmente, pelos próprios agressores, que nem sempre têm consciência de que se trata de um tipo de violência.

A negligência familiar contra as pessoas idosas é uma questão de relevância global, caracterizada pela falta de cuidados e apoio essencial, que são particularmente importantes em uma fase da vida em que as pessoas podem estar mais vulneráveis^(17)^. No contexto familiar, a negligência é frequentemente motivada por fatores como o estresse e a sobrecarga dos cuidadores^(18)^, combinados com a falta de apoio adequado por parte das políticas públicas e instituições de saúde. Estudos revelam que a negligência, seja física, emocional ou financeira, é uma das formas mais comuns de abuso em pessoas idosas, especialmente em países onde a tradição cultural e as expectativas sociais impõem aos familiares o papel de cuidadores principais^(19)^.

Em países em desenvolvimento, como o Brasil, o fenômeno da negligência familiar intensifica-se em virtude de fatores como o envelhecimento acelerado da população e as desigualdades socioeconômicas^(20)^. A negligência, muitas vezes invisível e subnotificada, compromete a qualidade de vida das pessoas idosas, privando-as de acesso a cuidados médicos, alimentação adequada e apoio emocional. Adicionalmente, fatores culturais e econômicos podem influenciar a prevalência do abuso e da negligência, com familiares enfrentando dificuldades financeiras que reduzem a disponibilidade de suporte adequado para seus membros idosos^(21)^.

Ainda no *Case 1*, ressalta-se que a realização de denúncias é essencial para evidenciar e combater a violência contra as pessoas idosas. A baixa taxa de denúncia é atribuída a fatores como o medo de represálias, a vergonha e a falta de conhecimento sobre os canais disponíveis para reportar o abuso. A Organização Mundial da Saúde (OMS) estima que apenas uma pequena fração dos casos de abuso contra as pessoas idosas é formalmente reportada, sugerindo que o problema é significativamente maior do que aparenta^(1)^.

Na Turquia, por exemplo, estudo com 161 médicos de família revelou que a maioria possui conhecimento limitado sobre abusos e negligência contra pessoas idosas, resultando em uma baixa taxa de notificações e denúncias. Essa subnotificação destaca a necessidade de políticas e sistemas de apoio que facilitem e incentivem as denúncias, além de treinar os profissionais de saúde e cuidadores para identificarem sinais de abuso e encorajarem as vítimas ou familiares a denunciarem^(22)^.

Além disso, as barreiras culturais podem influenciar a decisão de denunciar, como evidenciado na Índia e em alguns grupos culturais nos Estados Unidos, onde o valor atribuído às relações familiares ou o medo de estigmatização dificultam a abertura de processos formais contra os abusadores^(23)^. O fortalecimento de campanhas de conscientização e a criação de linhas de apoio seguras e confidenciais são medidas necessárias para superar essas barreiras e assegurar que os idosos tenham seus direitos garantidos e sejam protegidos de abusos^(24)^.

Essas iniciativas buscam transformar o ato de denunciar em um passo acessível e seguro para as pessoas idosas e seus familiares, além de sensibilizar a sociedade sobre a gravidade e as consequências da violência contra a pessoa idosa, ajudando a reduzir sua invisibilidade e a construir uma rede de proteção mais eficaz.

No *Case* 4, relativo ao desrespeito aos direitos da pessoa idosa, houve uma diferença significativa entre os grupos, com os estudantes do grupo de intervenção demonstrando maior percepção sobre a necessidade de prevenção e respeito aos direitos dos idosos em espaços públicos (p<0,001). Esse resultado sugere que a intervenção foi eficaz não apenas em transferir conhecimento, mas em fomentar uma postura ética diante de situações cotidianas, reconhecendo a cidadania dos idosos.

Os direitos da pessoa idosa têm sido um tema debatido e abordado por diversas organizações, incluindo a OMS e a Organização das Nações Unidas (ONU)^(1)^. Apesar dos avanços, a efetividade das políticas varia amplamente entre os países. Em algumas regiões, como a Europa, há uma legislação mais robusta e mecanismos de fiscalização para proteger os idosos contra a negligência e outros abusos. Por outro lado, em muitas nações em desenvolvimento, a falta de recursos e de políticas específicas impede que os direitos das pessoas idosas sejam garantidos e respeitados de forma eficaz. A pandemia da COVID-19 também expôs vulnerabilidades críticas, especialmente em instituições de longa permanência, onde a negligência se mostrou fatal em muitos casos por causa da falta de protocolos de segurança e cuidado adequados^(1-4)^.

No Brasil, as políticas públicas para a população idosa têm avançado de maneira significativa, refletindo uma resposta às necessidades crescentes dessa faixa etária. A promulgação da Política Nacional do Idoso, em 1994, representou um marco inicial, assegurando direitos sociais fundamentais para as pessoas idosas e estabelecendo diretrizes para sua integração e valorização na sociedade. Em 1999, a Política Nacional de Saúde do Idoso reforçou esse compromisso ao definir responsabilidades específicas para a promoção da saúde e bem-estar dessa população, visando ao envelhecimento ativo e saudável^(25)^.

A criação do Estatuto da Pessoa Idosa^(5)^ consolidou ainda mais esses direitos, sendo este amplamente reconhecido como uma das maiores conquistas sociais do país. O estatuto ampliou as responsabilidades, tanto do Estado quanto da sociedade, na proteção e no atendimento às necessidades dos idosos, assegurando direitos em áreas como saúde, assistência social e proteção contra a violência e negligência^(5)^.

As ações educativas desempenham papel crucial na prevenção da violência contra a pessoa idosa, sendo reconhecidas como uma ferramenta poderosa para enfrentar o problema em nível mundial. Essas ações incluem a capacitação de profissionais de saúde, campanhas de conscientização pública e a inserção de conteúdos educacionais sobre o respeito aos idosos em currículos escolares. Em muitos países, como a Índia, essas estratégias foram implementadas para promover a empatia entre as gerações e sensibilizar a população para os sinais de abuso^(23)^.

A educação também visa preparar cuidadores e familiares para lidar com o envelhecimento e com as necessidades específicas das pessoas idosas, de forma a evitar situações de abuso e negligência que possam resultar do desconhecimento ou despreparo. Além disso, observa-se que a formação de equipes multidisciplinares e a introdução de programas de apoio à família são formas eficazes de prevenção^(21-22)^.

Outrossim, a OMS destaca a importância das ações educativas no contexto da Década do Envelhecimento Saudável 2020-2030, que incentiva o treinamento de profissionais da saúde para identificar e responder a casos de abuso, por meio de uma abordagem baseada em direitos humanos, garantindo que as pessoas idosas tenham acesso a ambientes seguros e respeitosos^(26-27)^.

Essas iniciativas educacionais, combinadas com políticas públicas adequadas, formam uma abordagem global necessária para a proteção e valorização dos direitos dos idosos, ajudando a construir sociedades mais inclusivas e livres da violência contra a pessoa idosa.

A efetividade da intervenção educativa na alteração de percepções e atitudes, observada nos grupos de intervenção, está em consonância com a literatura, que destaca a importância de métodos ativos de ensino na área da saúde e humanas para engajar os estudantes em processos de aprendizagem que vão além do conhecimento teórico e promovem uma compreensão intersetorial dos direitos humanos das pessoas idosas^(28)^.

Os métodos ativos de aprendizagem constituem modalidades de ensino inovadoras no contexto da educação na área da saúde, pois buscam articular teoria e prática, por meio de aprendizagem significativa, e proporcionam mudanças necessárias à implementação dos cuidados preventivos para diminuição da violência contra a pessoa idosa, fortalecendo a construção de saberes de modo interdisciplinar^(29)^.

Os achados, embora ressaltem o valor das intervenções que utilizam gamificação na formação de profissionais sensíveis às realidades da pessoa idosa, são específicos do contexto estudado e não devem ser generalizados para outras situações ou ambientes.

Os resultados obtidos contribuem para evidenciar a necessidade de se ampliar o uso de metodologias inovadoras e interativas, como a gamificação, em currículos universitários, assim como demonstrado em uma pesquisa sobre os benefícios da gamificação na Educação Médica^(30)^. Essas abordagens parecem eficazes para promover atitudes de proteção e respeito aos idosos, atuando como ferramenta de transformação social e prevenção da violência.

Este estudo apresenta algumas limitações que devem ser consideradas na interpretação dos resultados. Primeiramente, por se tratar de um ensaio clínico não randomizado, a ausência de randomização pode ter introduzido vieses de seleção que afetam a representatividade e a generalização dos resultados. Embora os grupos tenham sido pareados para minimizar essas diferenças, o método não assegura a eliminação completa de variáveis confundidoras.

Embora o questionário tenha sido validado por juízes, respostas mais alinhadas ao socialmente esperado podem ter sido influenciadas pela própria natureza do instrumento e pela conscientização dos participantes quanto ao objetivo da intervenção. Ademais, outra influência pode estar relacionada às características individuais dos participantes, como o curso e experiência prévia com pessoas idosas. Acrescenta-se ainda a limitação do tamanho amostral.

Este estudo contribui para o avanço do conhecimento científico ao demonstrar que a intervenção gamificada pode ser uma estratégia efetiva para modificar atitudes e percepções de universitários sobre a violência contra a pessoa idosa, promovendo maior conscientização e propensão a denunciar casos de negligência. No entanto, a ausência de melhoria significativa no conhecimento sugere a necessidade de outras intervenções educativas complementares. Além disso, o estudo indica a importância de pesquisas futuras, especialmente estudos longitudinais, para avaliar a sustentação dessas mudanças ao longo do tempo.

## Conclusão

Este estudo evidenciou a importância das intervenções educativas gamificadas no desenvolvimento de atitudes protetoras e no aumento da percepção sobre a violência contra a pessoa idosa entre universitários. Por meio das ações educativas pautadas no processo de gamificação, foi possível envolver os participantes em um processo ativo, que promoveu uma aprendizagem mais prática e aplicada. Embora não tenha gerado melhorias significativas no conhecimento teórico, ela aumentou a conscientização dos participantes sobre a gravidade da violência contra pessoas idosas. Os resultados sugerem que metodologias ativas, como a gamificação, são eficazes para estimular mudanças de comportamento e fomentar a empatia e o respeito pelos direitos das pessoas idosas.

Conclui-se que, no contexto deste estudo, práticas educativas interativas e sensíveis às necessidades das pessoas idosas podem ajudar a formar profissionais mais conscientes sobre a violência contra a pessoa idosa. No entanto, esses achados são específicos ao ambiente estudado e não devem ser generalizados para outros contextos.

# Translation: Efecto de una intervención educativa para estudiantes universitarios sobre la violencia contra las personas mayores utilizando la gamificación: ensayo clínico no aleatorizado*

**DOI:** 10.1590/1518-8345.7874.4661

**Authors:** Juliana Ribeiro da Silva Vernasque, Miriam Fernanda Sanches Alarcon, Daiana Bonfim, Paula Sales Rodrigues, Eduardo Federighi Baisi Chagas, Maria José Sanches Marin

**Affiliations:** 1 Faculdade de Medicina de Marília, Educação em Ciências da Saúde, Marília, SP, Brasil.; 2 Universidade Estadual Paulista, Departamento de Enfermagem, Botucatu, SP, Brasil.; 3 Becaria de la Coordenação de Aperfeiçoamento de Pessoal de Nível Superior (CAPES), Brasil.; 4 Universidade Estadual do Norte do Paraná, Departamento de Enfermagem, Bandeirantes, PR, Brasil.; 5 Faculdade Israelita de Ciências da Saúde Albert Einstein, Centro de Estudos, Pesquisa e Prática APS e Redes, São Paulo, SP, Brasil.; 6 Faculdade de Medicina de Marília, Estatística, Marília, SP, Brasil.; 7 Faculdade de Medicina de Marília, Enfermagem em Saúde Coletiva, Marília, SP, Brasil.

**Keywords:** Anciano, Violencia, Universidades, Abuso de Ancianos, Actitud, Conocimiento

## Abstract

evaluar el impacto de una intervención educativa gamificada sobre violencia contra las personas mayores en el conocimiento, percepción y actitud de estudiantes universitarios.

ensayo clínico no aleatorizado con 44 estudiantes universitarios de ciencias de la salud, humanidades y ciencias exactas, 22 en el grupo de intervención y 22 en el grupo de control. El grupo de intervención participó en actividades híbridas de gamificación aplicadas al tema de la violencia contra las personas mayores. La evaluación se realizó mediante *cases* temáticos validados por expertos, con análisis estadístico mediante pruebas Chi-cuadrado y *t* de Student.

la intervención promovió diferencias significativas entre los grupos en cuanto a actitudes y percepciones sobre la violencia contra las personas mayores. Los estudiantes del grupo de intervención eran más propensos a denunciar casos de abandono y eran más conscientes de cómo prevenir el maltrato en los espacios públicos.

la intervención gamificada fue eficaz para estimular las actitudes protectoras y la percepción ética de la violencia contra las personas mayores, pero no hubo una asociación significativa con la variable conocimiento. Se necesitan estudios más exhaustivos que puedan aportar pruebas complementarias a los resultados de este estudio.

## Destacados

**(1)** Los universitarios son más propensos a denunciar la negligencia familiar. **(2)** Hay una mayor concienciación sobre los derechos de las personas mayores en los espacios públicos. **(3)** La intervención favorece la concienciación práctica sobre el maltrato a las personas mayores. **(4)** Los enfoques innovadores son eficaces para formar profesionales más empáticos. **(5)** Una intervención gamificada promueve actitudes protectoras hacia las personas mayores.

## Introducción

La violencia contra las personas mayores, caracterizada por acciones u omisiones que causan daño o sufrimiento a esta población, es un problema reconocido mundialmente^(1)^. Se estima que, por cada caso denunciado, otros cinco quedan sin denunciar, lo que pone de manifiesto la falta de denuncias y la invisibilidad del problema^(2)^.

La violencia contra los ancianos adopta diversas formas (abuso físico, psicológico, sexual, financiero y negligencia), cada una de las cuales tiene graves repercusiones y requiere enfoques intersectoriales para identificarla y combatirla. En particular, la mayoría de los agresores son miembros de la familia, lo que dificulta la denuncia y la intervención^(3)^. El reconocimiento de la violencia contra las personas mayores sigue siendo tardío e inadecuado entre las distintas áreas del conocimiento, y se considera un problema “subfinanciado, subexaminado e subreconocido”^(4)^.

Identificar y denunciar las situaciones en las que las personas mayores sufren violencia es un proceso complejo y cauteloso. Además, existe un desconocimiento entre los distintos sectores de la sociedad y entre las propias personas mayores sobre sus derechos y el significado de la violencia^(5)^.

Los estudios demuestran que las acciones educativas pueden ser eficaces para prevenir el maltrato contra las personas mayores. Un estudio transversal realizado en los Estados Unidos de América (EE.UU.) con 2.150 ancianos coreano-americanos analizó la prevalencia y los factores asociados al maltrato físico, emocional y financiero, y concluyó que se necesitan estrategias preventivas e intervenciones educativas contextualizadas^(6)^. En Israel, un estudio con 145 cirujanos ortopédicos demostró que los conocimientos y las actitudes más positivas se asociaban a un aumento de la identificación y la notificación de casos, lo que subraya la importancia de formarse en la detección y el tratamiento del maltrato a las personas mayores desde el inicio de su carrera^(7)^. Además, se ha demostrado que la formación en línea en una universidad de los EE.UU.^(8)^ mejora significativamente los conocimientos de los estudiantes sobre la interacción adecuada con las personas mayores víctimas de malos tratos. Se encontraron resultados similares entre los enfermeros iraníes^(9)^, ya que los niveles más altos de educación se asociaron con mejores prácticas asistenciales, aunque barreras como la carga de trabajo y la falta de formación siguen dificultando la aplicación de los conocimientos.

En este contexto, se entiende que las acciones educativas interdisciplinarias que promueven el aprendizaje significativo son esenciales para estimular no sólo el conocimiento, sino también las percepciones y actitudes de protección hacia la población anciana. Al involucrar a los estudiantes en una formación gamificada que integre valores y habilidades prácticas, se espera fomentar una comprensión más profunda y actitudes de respeto y protección, necesarias para abordar esta compleja problemática.

La gamificación es el uso de elementos y técnicas de diseño de juegos en la educación, la sanidad y el mundo empresarial. En el contexto educativo, la gamificación, especialmente cuando se combina con el aprendizaje colaborativo, contribuye al desarrollo de competencias transversales como la creatividad, la resolución de problemas y el trabajo en equipo, así como a mejorar el rendimiento académico y la motivación de los estudiantes^(10)^.

Así, la pregunta de investigación planteada es: ¿mejoran los jóvenes universitarios entrenados en el reconocimiento de la violencia contra las personas mayores a través de la gamificación sus conocimientos, actitud y percepción, resultando en una mayor responsabilidad que los universitarios no entrenados?

El objetivo de este estudio es evaluar el impacto de una intervención educativa gamificada sobre la violencia contra las personas mayores en el conocimiento, la percepción y la actitud de los estudiantes universitarios.

## Método

### Diseño del estudio

Se trata de un ensayo clínico no aleatorizado. Para mantener el rigor metodológico, se utilizó el protocolo *Standards for QUality Improvement Reporting Excellence in Education* (SQUIRE 2.0) como herramienta de apoyo para el desarrollo del estudio^(11)^.

Se optó por un diseño no aleatorio debido a la naturaleza del estudio y a las características del contexto de aplicación^(12)^. Ante la dificultad de asignar aleatoriamente a los participantes a los grupos de intervención, los responsables por el estudio optaron por involucrar a estudiantes universitarios que estuvieran disponibles y dispuestos a participar, sin necesidad de una división aleatoria entre los grupos. Este enfoque permitió aplicar una intervención educativa en un entorno real, proporcionando un análisis más flexible y práctico del impacto sobre los conocimientos, percepciones y actitudes de los participantes.

### Local de la investigación

El lugar de la investigación fue un municipio de tamaño medio situado en el Centro-Oeste del estado de São Paulo (Brasil), con una población estimada de 235.234 habitantes^(13)^. Este municipio cuenta con un gran complejo de instituciones de enseñanza superior (IES), tanto públicas como privadas, que ofrecen cursos en diferentes áreas del conocimiento. Para este estudio, se incluyeron cuatro IES, dos públicas y dos privadas, con el fin de involucrar estudiantes universitarios de las áreas de ciencias de la salud, ciencias exactas y humanidades.

### Período

El estudio se llevó a cabo entre diciembre de 2022 y abril de 2024.

### Población

Los participantes de este estudio fueron estudiantes universitarios de las áreas de salud, humanidades y ciencias exactas, seleccionados de manera no aleatoria, considerando el contexto y la naturaleza de la intervención educativa. Se formaron dos grupos: el primero (grupo intervención), compuesto por jóvenes universitarios que participaron en la intervención educativa; y el segundo (grupo de control), compuesto por jóvenes universitarios que no participaron en la intervención educativa. Durante el período de estudio, estos participantes mantuvieron sus actividades académicas habituales, sin participar en actividades relacionadas con el tema de la intervención ni realizar cambios específicos en su rutina académica.

### Criterios de selección

El grupo de intervención fue reclutado en diciembre de 2022 para participar durante un año, hasta diciembre de 2023. Inicialmente, se reunieron 40 jóvenes universitarios para seleccionar a los participantes (grupo de intervención): estudiantes de las carreras de Enfermería, Medicina, Derecho, Terapia Ocupacional, Fisioterapia, Ingeniería Civil, Psicología y Pedagogía. Los estudiantes fueron seleccionados en función de su interés en participar en la intervención educativa. Veintidós estudiantes, de los siguientes cursos Enfermería, Medicina, Derecho, Terapia Ocupacional y Fisioterapia, participaron en todo el proceso.

Los miembros del grupo de control se seleccionaron por conveniencia, se reclutaron en marzo de 2024 y no se llevó a cabo ninguna intervención. Para la composición de los grupos, las invitaciones se realizaron con la colaboración de profesores y coordinadores de curso de las instituciones participantes. El diseño de la intervención fue controlado; los grupos se constituyeron por emparejamiento, garantizando una composición rigurosa y abierta^(12)^.

Los criterios de inclusión en el estudio fueron los siguientes: ser estudiante universitario; estar matriculado en una de las cuatro instituciones de enseñanza superior participantes; y, en el caso del grupo de intervención, estar disponible para participar en actividades educativas a lo largo de un año. Se excluyeron del estudio los estudiantes universitarios que no pertenecían a los programas de ciencias de la salud, humanidades o ciencias exactas; los estudiantes que no tenían acceso a dispositivos digitales o a Internet; y las personas que no rellenaron el instrumento de recogida de datos.

### Intervención

La intervención educativa se desarrolló a lo largo de un año, con cuatro encuentros presenciales, intercalados con encuentros online, que sumaron un total de 50 horas. El proceso educativo utilizó la gamificación, a través de conferencias, clases de diálogo expositivo y talleres, en los que se animó a los alumnos a reflexionar sobre la violencia contra las personas mayores, a partir de sus propias experiencias, así como sobre las posibilidades de desarrollar juegos con vistas a promover la salud de las personas mayores y prevenir la violencia. Las actividades contaron con el apoyo de profesores e investigadores sobre el tema de la “Violencia contra las personas mayores” y de un especialista en juegos.

Se entiende que el uso de las tecnologías de la información en los procesos educativos, en todos los campos del conocimiento, puede potenciar el aprendizaje significativo y contribuir a la construcción de conocimientos prácticos y al cambio de actitudes.

En la primera reunión presencial, hubo una presentación sobre el tema “Violencia contra las personas mayores”, que abarcó conceptos importantes, aspectos epidemiológicos y los resultados de estudios realizados en el propio municipio, que ponen de relieve los problemas a los que se enfrenta este sector de la población^(14)^.

A continuación, se realizó una presentación dialogada sobre los principios y etapas de la gamificación, destacando la intersección entre los dos temas: gamificación y violencia contra las personas mayores.

En la dinámica del proyecto, los estudiantes se dividieron en grupos en función de sus afinidades para crear propuestas de juego sobre el tema de la violencia contra las personas mayores. En cada nuevo encuentro, los grupos presentaban los avances de su trabajo, seguido de un espacio de discusión para que los demás participantes pudieran analizar y evaluar los proyectos en desarrollo, ofreciendo sugerencias para mejorar las propuestas. De este modo, las propuestas de cada grupo se fueron construyendo y perfeccionando en el seno de los pequeños grupos y con la contribución de todos los participantes.

Al final, se desarrollaron seis proyectos con gamificación, que permitieron a los alumnos participantes implicarse permanentemente durante todo el periodo, tanto en relación con el tema como en la propia construcción de un proyecto que pudiera tener aplicación práctica en la sociedad.

### Variables del estudio

Para caracterizar la muestra, se recogieron datos sobre la edad, el sexo y la licenciatura. Para evaluar la eficacia de la intervención, el resultado primario fue el porcentaje de respuestas correctas a los *cases* propuestos. Se prepararon cinco *cases*, cada uno destinado a abordar una forma específica de violencia contra las personas mayores, lo que permitió un amplio análisis de los distintos tipos de maltrato.

Para la validación de los *cases* por parte de los jueces, se utilizó una escala Likert, que permitía a los evaluadores expresar el grado de acuerdo con la adecuación de cada pregunta a la evaluación pretendida.

Este proceso buscó garantizar que las preguntas captaran con precisión las percepciones, conocimientos técnicos y actitudes de los alumnos frente a las diferentes situaciones de violencia que enfrentan las personas mayores en diversos contextos. Además, se habilitó un espacio para sugerencias y opiniones sobre determinados contenidos cuando el evaluador lo consideró necesario. La validación del contenido de los *cases* se llevó a cabo con la participación de 11 jueces invitados expertos en el campo de la geriatría y gerontología o en procesos de evaluación del aprendizaje, que firmaron el Formulario de Consentimiento Libre e Informado.

En cuanto a las características de los jueces, había nueve mujeres y dos hombres; edad media de 53 años; formación profesional, tres licenciados en Enfermería, cuatro en Medicina, dos en Psicología, uno en Ciencias Farmacéuticas y uno en Trabajo Social. La duración media de la formación fue de 29,54 años y la duración media de actividad en el trabajo actual fue de 26,54 años. La validación tuvo lugar en dos momentos distintos, teniendo en cuenta que los jueces hicieron sugerencias pertinentes que se añadieron al instrumento de evaluación.

Para analizar el nivel de acuerdo entre los jueces se utilizó el coeficiente de validez del contenido (CVC)^(15)^. El CVC es un indicador recomendado para calcular el nivel de consenso, mediante el cálculo del valor medio atribuido por los jueces a cada ítem del instrumento, expresado en términos porcentuales. Se calculó el CVC individual para cada pregunta y la media de todos ellos para el CVC global, considerándose válidos los ítems con porcentajes iguales o superiores al 80%^(16)^. Para el instrumento con los cinco *cases* y las seis preguntas de cada *case*, se calculó el CVC, que fue de 0,94 para ambos momentos.

Los *cases* validados se introdujeron en un formulario de *Google Forms* y luego se enviaron por *WhatsApp* a los participantes, tanto para el grupo de intervención como para el grupo de control. El *Case 1* se centra en la negligencia familiar, examinando si los estudiantes pudieron identificar signos de descuido y omisión en el cuidado de los ancianos. El *Case 2* trata de la violencia financiera, evaluando la percepción de los alumnos a respecto de la explotación económica y el control indebido de los bienes de los ancianos. En el *Case 3*, se hizo hincapié en la violencia física en una institución de larga estancia, tratando de evaluar cómo reconocer los signos de agresión física. El *Case 4* trató de la falta de respeto a los derechos de las personas mayores en el transporte público, incitando a los alumnos a reflexionar sobre actitudes de ciudadanía y respeto. Por último, el *Case 5* exploró la violencia sexual, centrándose en la identificación de los abusos y las consecuencias de las relaciones abusivas.

Para cada *case*, se formularon seis preguntas para evaluar los conocimientos, las percepciones y las actitudes de los participantes. Las preguntas uno y seis estaban destinadas a evaluar la percepción de los estudiantes sobre la violencia contra los ancianos en la sociedad; las preguntas dos y tres estaban destinadas a calibrar los conocimientos; y las preguntas cuatro y cinco estaban destinadas a evaluar las actitudes. Cada pregunta se respondía con opciones que iban desde “sí”, “no”, “no puedo asegurarlo”, “conocimientos insuficientes” y “sin posición definida”. Sin embargo, para calcular el porcentual de aciertos solo se tuvo en cuenta la suma de respuestas correctas.

### Análisis de los datos

Las variables cualitativas se describen mediante una distribución de frecuencias absolutas (N) y relativas (%). Para analizar la diferencia en la distribución de frecuencias, se utilizó la prueba de Chi-cuadrado para la proporción. Para analizar la relación entre las variables cualitativas y el grupo, se utilizó la prueba de asociación Chi-cuadrado. Para comparar las medias se utilizó la prueba *t* de Student para muestras independientes tras comprobar la homogeneidad de las varianzas mediante la prueba de Levene. El nivel de significación adoptado fue del 5% y los datos se analizaron con el programa SPSS (versión 27.0).

### Aspectos éticos

La investigación fue aprobada por el Comité de Ética de la Investigación, con el informe n.º 5.144.186, y todos los participantes firmaron el Formulario de Consentimiento Libre e Informado.

## Resultados

Había 22 estudiantes en el grupo de intervención y 22 en el grupo de control. La edad media del grupo de intervención era de 23,00 ± 2,11 años; la edad media del grupo de control era de 24,04 ± 2,11 años. Los grupos de intervención y de control eran homogéneos en cuanto a género y programas de estudios (Tabla 1).

**Tabla 1-:** Distribución de los participantes, grupo de intervención y grupo de control, según sexo y licenciatura (n = 44). Marília, SP, Brasil, 2024

		**Grupo**				**Total**		valor *p* a*	**valor *p* b** ^†^
		**Intervención (n=22)**		**Control (n=22)**					
		**N**	**%**	**N**	**%**	**N**	**%**		
**Sexo**	Masculino	5	22,7%	5	22,7%	10	22,7%	<0,001 ^‡^	0,999
	Femenino	17	77,3%	17	77,3%	34	77,3%		
**Licenciatura**	Medicina	10	45,5%	12	54,5%	22	50,0%	<0,001 ^‡^	0,904
	Enfermería	8	36,4%	5	22,7%	13	29,5%		
	Terapia Ocupacional	1	4,5%	1	4,5%	2	4,5%		
	Derecho	2	9,1%	2	9,1%	4	9,1%		
	Fisioterapia	1	4,5%	2	9,1%	3	6,8%		

*valor *p* a = valor *p* calculado mediante la prueba de Chi-cuadrado para la proporción. Esta prueba comprueba si existe una diferencia en la distribución de proporciones en el conjunto de las categorías de respuesta; ^†^valor *p* b = valor *p* calculado mediante la prueba de Chi-cuadrado para asociación. Esta prueba comprueba si existe una diferencia en la distribución de proporciones entre el grupo de intervención y el de control; ^‡^Indica un efecto significativo para valor *p* a ≤ 0,050 mediante la prueba de Chi-cuadrado para la proporción

La Tabla 2 muestra que en el *Case 1* hubo diferencia estadísticamente significativa entre el grupo de intervención y el de control con relación a la pregunta 5 − “*Frente a la situación de la Sra. Catarina, ¿denunciaría al dial 100?*” − que evaluaba la actitud del universitario frente a una situación de violencia contra ancianos.

**Tabla 2-:** Distribución de los participantes, grupo de intervención y grupo de control, según la respuesta a cada una de las preguntas del *Case1*(n = 44). Marília, SP, Brasil, 2024

		**Grupo**				**Total**		valor *p* a*	**valor *p* b** ^†^
		**Intervención (n=22)**		**Control (n=22)**					
		**N**	**%**	**N**	**%**	**N**	**%**		
Case 1 **Q** ^§^ **1**	Si	18	81,8%	17	77,3%	35	79,5%	<0,001 ^‡^	0,811
	No	1	4,5%	4	18,2%	5	11,4%		
	No puedo asegurarlo	3	13,6%	1	4,5%	4	9,1%		
Case 1 **Q** ^§^ **2**	Si	22	100,0%	22	100,0%	44	100,0%	NA ^||^	NA ^||^
Case 1 **Q** ^§^ **3**	Si	21	95,5%	21	95,5%	42	95,5%	<0,001 ^‡^	0,999
	Conocimientos insuficientes	1	4,5%	1	4,5%	2	4,5%		
Case 1 **Q** ^§^ **4**	Si	20	90,9%	21	95,5%	41	93,2%	<0,001 ^‡^	0,554
	Sin posición definida	2	9,1%	1	4,5%	3	6,8%		
Case 1 **Q** ^§^ **5**	Si	22	100,0%	12	54,5%	34	77,3%	<0,001 ^‡^	<0,001 ^¶^
	No	0	0,0%	3	13,6%	3	6,8%		
	Sin posición definida	0	0,0%	7	31,8%	7	15,9%		
Case 1 **Q** ^§^ **6**	Si	21	95,5%	20	90,9%	41	93,2%	<0,001 ^‡^	0,307
	No	1	4,5%	0	0,0%	1	2,3%		
	No puedo asegurarlo	0	0,0%	2	9,1%	2	4,5%		

*valor *p* a = valor *p* calculado mediante la prueba de Chi-cuadrado para la proporción. Esta prueba comprueba si existe una diferencia en la distribución de proporciones en las categorías de respuesta en total; **†**valor *p* b = valor *p* calculado por la prueba Chi-cuadrado para asociación. Esta prueba comprueba si existe una diferencia en la distribución de proporciones entre el grupo de intervención y el de control; ‡Indica un efecto significativo para el valor *p* a ≤ 0,050 mediante la prueba de Chi-cuadrado para la proporción; ^§^Q = Indica la pregunta de cada *Case*; ^||^NA = Indica que no fue posible calcular el valor *p*, ya que todos dieron la misma respuesta; ^¶^Indica un efecto significativo para el valor *p* b ≤ 0,050 mediante la prueba de Chi-cuadrado para la asociación

En los *Cases* 2, 3, y 5, no hubo diferencias estadísticamente significativas entre los grupos de intervención y de control.

La Tabla 3 muestra que, en el *Case 4*, hubo una diferencia estadísticamente significativa entre los grupos de intervención y de control con respecto a la pregunta 6 − “¿Cree que se pueden prevenir situaciones como ésta?*“* − que evaluaba la percepción de los estudiantes universitarios sobre cómo prevenir la violencia contra las personas mayores en lo que se refiere a sus derechos en la sociedad.

**Tabla 3-:** Distribución de los participantes, grupo de intervención y grupo de control, según sus respuestas a cada una de las preguntas del *Case4* (n = 44). Marília, SP, Brasil, 2024

		**Grupo**				**Total**		valor *p* a*	**valor *p* b** ^†^
		**Intervención (n=22)**		**Control (n=22)**					
		N	**%**	**N**	**%**	**N**	**%**		
Case 4 **Q** ^§^ **1**	Si	17	77,3%	21	95,5%	38	86,4%	<0,001 ^‡^	0,078
	No	4	18,2%	1	4,5%	5	11,4%		
	No puedo asegurarlo	1	4,5%	0	0,0%	1	2,3%		
Case 4 **Q** ^§^ **2**	Si	3	13,6%	0	0,0%	3	6,8%	<0,001 ^‡^	0,106
	No	18	81,8%	20	90,9%	38	86,4%		
	Conocimientos insuficientes	1	4,5%	2	9,1%	3	6,8%		
Case 4 **Q** ^§^ **3**	Si	17	77,3%	17	77,3%	34	77,3%	<0,001 ^‡^	0,675
	No	3	13,6%	1	4,5%	4	9,1%		
	Conocimientos insuficientes	2	9,1%	4	18,2%	6	13,6%		
Case 4 **Q** ^§^ **4**	Si	1	4,5%	0	0,0%	1	2,3%	<0,001 ^‡^	0,209
	No	21	95,5%	21	95,5%	42	95,5%		
	Sin posición definida	0	0,0%	1	4,5%	1	2,3%		
Case 4 **Q** ^§^ **5**	Si	17	77,3%	14	63,6%	31	70,5%	<0,001 ^‡^	0,463
	No	1	4,5%	3	13,6%	4	9,1%		
	Sin posición definida	4	18,2%	5	22,7%	9	20,5%		
Case 4 **Q** ^§^ **6**	Si	1	4,5%	20	90,9%	21	47,7%	<0,001 ^‡^	<0,001 ^||^
	No	21	95,5%	0	0,0%	21	47,7%		
	No puedo asegurarlo	0	0,0%	2	9,1%	2	4,5%		

*valor *p* a = valor *p* calculado mediante la prueba de Chi-cuadrado para la proporción. Esta prueba comprueba si existe una diferencia en la distribución de proporciones en las categorías de respuesta en total; **†**valor *p* b = valor *p* calculado por la prueba de Chi-cuadrado para asociación. Esta prueba comprueba si existe una diferencia en la distribución de proporciones entre el grupo de intervención y el control; ^‡^Indica un efecto significativo para el valor *p* a ≤ 0,050 mediante la prueba de Chi-cuadrado para la proporción; ^§^Q = Indica la pregunta de cada *Case*; ^||^Indica un efecto significativo para el valor *p* b ≤ 0,050 mediante la prueba de Chi-cuadrado para la asociación

Cuando se agrupan las preguntas de los cinco *cases*, se observa que había una diferencia significativa entre el grupo de intervención para las preguntas 5 y 6, en las que la pregunta 5 evaluaba las actitudes y la pregunta 6 las percepciones de los estudiantes universitarios (Tabla 4).

**Tabla 4-:** Distribución de los participantes, grupo de intervención y grupo de control, según el grupo de respuesta (n = 44). Marília, SP, Brasil, 2024

**Variable**	**Grupo**	**Media**	DE*	**valor** ***p*** ^†^
**Q** ^‡^ **1% aciertos**	Intervención	67,273	29,3066	0,676
	Control	63,636	28,0383	
**Q** ^‡^ **2% aciertos**	Intervención	81,818	8,5280	0,060
	Control	77,273	7,0250	
**Q** ^‡^ **3% aciertos**	Intervención	93,636	11,3580	0,338
	Control	90,000	13,4519	
**Q** ^‡^ **4% aciertos**	Intervención	75,455	10,5683	0,504
	Control	72,727	15,7908	
**Q** ^‡^ **5% aciertos**	Intervención	57,273	9,3513	<0,001
	Control	41,818	15,0036	
**Q** ^‡^ **6% aciertos**	Intervención	79,091	4,2640	0.035 ^§^
	Control	89,091	21,1365	
**Total de aciertos Cuestiones%**	Intervención	75,758	6,6812	0,177 ^§^
	Control	72,424	9,2110	

*DE = Indica la desviación estándar; ^†^valor *p* = valor *p* calculado mediante la prueba *t* de Student; ^‡^Q = Indica la pregunta de cada *Case*; ^§^Indica la diferencia significativa entre grupos mediante la prueba *t* de Student para valor *p* ≤ 0,050

## Discusión

Este estudio investigó el efecto de una intervención educativa sobre los conocimientos, percepciones y actitudes de los estudiantes universitarios respecto a la violencia contra las personas mayores. Los resultados mostraron diferencias significativas entre el grupo de intervención y el grupo de control en aspectos relacionados con actitudes y percepciones, especialmente en cuestiones que requerían posturas más activas, como la denuncia de casos de negligencia y la prevención de situaciones de maltrato.

En el *Case 1*, que trataba de la negligencia familiar, los participantes del grupo de intervención fueron más propensos a denunciar la situación, lo que sugiere que la intervención promovió la concienciación práctica y ética sobre el rol de los estudiantes y futuros profesionales frente al maltrato (*p* < 0,001). Esta diferencia entre los grupos refuerza la eficacia de las estrategias educativas que integran la gamificación e implican la simulación de escenarios prácticos, ya que potencian el desarrollo de actitudes protectoras ante situaciones de negligencia. El abandono familiar de las personas mayores es un tipo de violencia muy sutil, a menudo trivializado, “subreconocido”^(4)^ por la sociedad y especialmente por los propios autores, que no siempre son conscientes de que se trata de un tipo de violencia.

El abandono familiar de las personas mayores es un problema de relevancia mundial, caracterizado por la falta de cuidados y apoyo esenciales, que es especialmente importante en una etapa de la vida en la que las personas pueden ser más vulnerables^(17)^. En el contexto familiar, el abandono suele estar motivado por factores como el estrés y la sobrecarga de los cuidadores^(18)^, combinados con la falta de apoyo adecuado por parte de las políticas públicas y las instituciones sanitarias. Los estudios demuestran que la negligencia, ya sea física, emocional o económica, es una de las formas más comunes de maltrato a las personas mayores, sobre todo en países donde la tradición cultural y las expectativas sociales imponen el papel de cuidador principal a los miembros de la familia^(19)^.

En los países en desarrollo, como Brasil, el fenómeno del abandono familiar se está intensificando debido a factores como el envejecimiento acelerado de la población y las desigualdades socioeconómicas^(20)^. El abandono, a menudo invisible y poco denunciado, pone en peligro la calidad de vida de las personas mayores, privándolas de acceso a atención médica, alimentación adecuada y apoyo emocional. Además, los factores culturales y económicos pueden influir en la prevalencia del maltrato y la negligencia, ya que los familiares se enfrentan a dificultades económicas que reducen la disponibilidad de un apoyo adecuado para sus miembros mayores^(21)^.

El *Case 1* también subraya que denunciar los malos tratos es esencial para poner de relieve y combatir la violencia contra las personas mayores. El bajo índice de denuncias se atribuye a factores como el miedo a las represalias, la vergüenza y el desconocimiento de los canales disponibles para denunciar los malos tratos. La Organización Mundial de la Salud (OMS) estima que sólo se denuncia formalmente una pequeña parte de los casos de maltrato contra las personas mayores, lo que sugiere que el problema es mucho mayor de lo que parece^(1)^.

En Turquía, por ejemplo, un estudio realizado entre 161 médicos de familia reveló que la mayoría tiene un conocimiento limitado del maltrato y la negligencia contra las personas mayores, lo que se traduce en una baja tasa de notificación y denuncia. Este subregistro pone de manifiesto la necesidad de políticas y sistemas de apoyo que faciliten y fomenten la denuncia, así como de formar a los profesionales de salud y cuidadores para que identifiquen los signos de maltrato y animen a las víctimas o familiares a denunciarlo^(22)^.

Además, las barreras culturales pueden influir en la decisión de denunciar, como se evidencia en India y algunos grupos culturales de los Estados Unidos, donde el valor que se da a las relaciones familiares o el miedo a la estigmatización hacen difícil presentar denuncias formales contra los abusadores^(23)^. El fortalecimiento de las campañas de concienciación y la creación de líneas telefónicas de ayuda seguras y confidenciales son medidas necesarias para superar estas barreras y asegurar que las personas mayores tengan garantizados sus derechos y estén protegidas contra el abuso^(24)^.

Estas iniciativas buscan transformar el acto de denuncia en un paso accesible y seguro para las personas mayores y sus familias, además de concienciar a la sociedad sobre la gravedad y las consecuencias de la violencia contra las personas mayores, ayudando a reducir su invisibilidad y construir una red de protección más efectiva.

En el *Case 4*, relativo al irrespeto a los derechos de las personas mayores, se encontró una diferencia significativa entre los grupos, demostrando los estudiantes del grupo de intervención una mayor conciencia de la necesidad de prevención y respeto a los derechos de las personas mayores en los espacios públicos (*p* < 0,001). Este resultado sugiere que la intervención fue efectiva no sólo en la transferencia de conocimiento, sino en el fomento de una postura ética en situaciones cotidianas, reconociendo la ciudadanía de los mayores.

Los derechos de las personas mayores han sido un tema debatido y abordado por varias organizaciones, incluidas la OMS y las Naciones Unidas (ONU)^(1)^. A pesar de los avances, la eficacia de las políticas varía mucho según los países. En algunas regiones, como Europa, existen leyes y mecanismos de aplicación más sólidos para proteger a las personas mayores del abandono y otros abusos. Por otra parte, en muchos países en desarrollo, la falta de recursos y de políticas específicas impide que los derechos de las personas mayores se garanticen y respeten efectivamente. La pandemia de COVID-19 también ha expuesto vulnerabilidades críticas, especialmente en los centros de atención a largo plazo, donde la negligencia ha resultado fatal en muchos casos debido a la falta de protocolos de seguridad y atención adecuados^(1-4)^.

En Brasil, las políticas públicas para la población anciana han avanzado significativamente, reflejando una respuesta a las crecientes necesidades de ese grupo etario. La promulgación de la Política Nacional de las Personas Mayores en 1994 representó un hito inicial, al garantizar los derechos sociales fundamentales de las personas mayores y establecer directrices para su integración y valoración en la sociedad. En 1999, la Política Nacional de Salud para las Personas Mayores reforzó este compromiso al definir responsabilidades específicas para promover la salud y el bienestar de esta población, apuntando al envejecimiento activo y saludable^(25)^.

La creación del Estatuto de las Personas Adultas Mayores^(5)^ consolidó aún más estos derechos, y esto es ampliamente reconocido como uno de los mayores logros sociales del país. El estatuto amplió las responsabilidades tanto del Estado como de la sociedad en la protección y satisfacción de las necesidades de las personas mayores, garantizando derechos en áreas como la salud, la asistencia social y la protección contra la violencia y el abandono^(5)^.

Las acciones educativas desempeñan un papel crucial en la prevención de la violencia contra las personas mayores y se reconocen como una herramienta poderosa para abordar el problema en todo el mundo. Estas acciones incluyen la formación de profesionales de salud, campañas de concienciación ciudadana y la inclusión de contenidos educativos sobre el respeto a las personas mayores en los currículos escolares. En muchos países, como en India, se han implementado estas estrategias para promover la empatía intergeneracional y generar conciencia sobre los signos de abuso^(23)^.

La educación también tiene como objetivo preparar a los cuidadores y familiares para afrontar el envejecimiento y las necesidades específicas de las personas mayores, con el fin de evitar situaciones de abuso y negligencia que puedan derivar de la falta de conocimientos o preparación. Además, se observa que la formación de equipos multidisciplinarios y la introducción de programas de apoyo familiar son formas efectivas de prevención^(21-22)^. Asimismo, la OMS destaca la importancia de las acciones educativas en el contexto de la Década del Envejecimiento Saludable 2020-2030, que fomenta la capacitación de los profesionales de la salud para identificar y responder a los casos de maltrato a través de un enfoque basado en los derechos humanos, garantizando que las personas mayores tengan acceso a entornos seguros y respetuosos^(26-27)^. Estas iniciativas educativas, combinadas con políticas públicas adecuadas, conforman un enfoque global necesario para proteger y valorar los derechos de las personas mayores, ayudando a construir sociedades más inclusivas y libres de violencia contra las personas mayores.

La eficacia de la intervención educativa en el cambio de percepciones y actitudes, observada en los grupos de intervención, está en consonancia con la bibliografía, que destaca la importancia de los métodos activos de enseñanza en el ámbito de la salud y de las humanidades para involucrar a los estudiantes en procesos de aprendizaje que vayan más allá de los conocimientos teóricos y promuevan una comprensión intersectorial de los derechos humanos de las personas mayores^(28)^. Los métodos de aprendizaje activo son modalidades de enseñanza innovadoras en el contexto de la educación en salud, ya que buscan articular la teoría y la práctica a través del aprendizaje significativo y proporcionan los cambios necesarios para implementar cuidados preventivos para reducir la violencia contra las personas mayores, fortaleciendo la construcción del conocimiento de forma interdisciplinaria^(29)^. Aunque los hallazgos destaquen el valor de las intervenciones con gamificación en la formación de profesionales sensibles a las realidades de los ancianos, son específicos al contexto estudiado y no deben ser generalizados a otras situaciones o ambientes.

Los resultados obtenidos contribuyen a resaltar la necesidad de extender el uso de metodologías innovadoras e interactivas, como la gamificación, en los currículos universitarios, tal y como demostró un estudio sobre los beneficios de la gamificación en la educación médica^(30)^. Estos enfoques parecen ser eficaces para promover actitudes de protección y respeto hacia las personas mayores, actuando como herramientas de transformación social y prevención de la violencia.

Este estudio tiene algunas limitaciones que deben tenerse en cuenta al interpretar los resultados. En primer lugar, al tratarse de un ensayo clínico no aleatorizado, la falta de aleatorización puede haber introducido sesgos de selección que afecten a la representatividad y la naturaleza general de los resultados. Aunque los grupos se emparejaron para minimizar estas diferencias, el método no garantiza la eliminación completa de las variables que generan confusión.

Aunque el cuestionario fue validado por jueces, las respuestas más acordes con lo socialmente esperado pueden haberse visto influidas por la propia naturaleza del instrumento y el conocimiento de los participantes del objetivo de la intervención. Además, otra influencia puede estar relacionada con las características individuales de los participantes, como su curso de estudios y su experiencia previa con personas mayores. Además, el tamaño de la muestra era limitado.

Este estudio contribuye al avance del conocimiento científico al demostrar que la intervención gamificada puede ser una estrategia eficaz para cambiar las actitudes y percepciones de los estudiantes universitarios sobre la violencia contra las personas mayores, promoviendo una mayor concienciación y propensión a denunciar los casos de negligencia. Sin embargo, la falta de mejoras significativas en los conocimientos sugiere la necesidad de otras intervenciones educativas complementarias. Además, el estudio indica la importancia de futuras investigaciones, especialmente estudios longitudinales, para evaluar la sostenibilidad de estos cambios a lo largo del tiempo.

## Conclusión

Este estudio puso de manifiesto la importancia de las intervenciones educativas gamificadas para desarrollar actitudes protectoras y aumentar la concienciación sobre la violencia contra las personas mayores entre los estudiantes universitarios. A través de acciones educativas basadas en el proceso de gamificación, se consiguió involucrar a los participantes en un proceso activo que promovió un aprendizaje más práctico y aplicado. Aunque no generó mejoras significativas en los conocimientos teóricos, sí aumentó la concienciación de los participantes sobre la gravedad de la violencia contra las personas mayores. Los resultados sugieren que metodologías activas como la gamificación son eficaces para estimular cambios de comportamiento y fomentar la empatía y el respeto por los derechos de las personas mayores.

Se concluye que, en el contexto de este estudio, las prácticas educativas interactivas sensibles a las necesidades de las personas mayores pueden contribuir a formar profesionales más conscientes de la violencia contra las personas mayores. Sin embargo, estas conclusiones son específicas del entorno estudiado y no deben generalizarse a otros contextos.
