# Supplementary material for: Erratum: The effect of an educational intervention for university students on violence against the elderly using gamification: a non-randomized clinical trial
Source: Rev Lat Am Enfermagem. 2025 Oct 27;33:e4741. doi: 10.1590/1518-8345.0000.4741 (PMC12562685; doi:10.1590/1518-8345.0000.4741)
Supplement: Related texts

# Correction: Errata: Efeito de uma intervenção educativa para universitários sobre violência contra pessoas idosas por meio da gamificação: ensaio clínico não randomizado

**DOI:** 10.1590/1518-8345.0000.4742

**Published:** 2025

No artigo “Efeito de uma intervenção educativa para universitários sobre violência contra pessoas idosas por meio da gamificação: ensaio clínico não randomizado”, com número DOI: 10.1590/1518-8345.7874.4663, publicado no periódico Rev. Latino-Am. Enfermagem, 2025;33:e4663:

Na seção Resultados, no 4º parágrafo, página 6:

Onde se lia:

“Nos *Cases* 2, 3, e 5”

Leia-se:

“Nos *Cases* 2, 3, 4 e 5”

Na seção Resultados, no 5º parágrafo, página 6:

Onde se lia:

“Na Tabela 3, observa-se que, no *Case* 4, houve diferença significativamente estatística entre os grupos de intervenção e controle”

Leia-se:

“Na Tabela 3, observa-se que, no *Case* 4, não houve diferença significativamente estatística entre os grupos de intervenção e controle”

Na seção Resultados, na Tabela 3, página 6:

Onde se lia:

**Table:** 

		**Grupo**				**Total**		*p* -valor a*	***p* -valor b** ^†^
		**Intervenção (n=22)**		**Controle (n=22)**					
		N	**%**	**N**	**%**	**N**	**%**		
*Case* 4 Q ^§^ 6	Sim	1	4,5%	20	90,9%	21	47,7%	<0,001 ^‡^	<0,001 ^||^
	Não	21	95,5%	0	0,0%	21	47,7%		
	Não posso afirmar com certeza	0	0,0%	2	9,1%	2	4,5%		

Leia-se:

**Table:** 

		**Grupo**				**Total**		*p* -valor a*	***p* -valor b** ^†^
		**Intervenção (n=22)**		**Controle (n=22)**					
		N	**%**	**N**	**%**	**N**	**%**		
*Case* 4 Q ^§^ 6	Sim	21	95,5%	20	90,9%	41	93,2%	<0,001 ^‡^	<0,220
	Não	1	4,5%	0	0,0%	1	2,3%		
	Não posso afirmar com certeza	0	0,0%	2	9,1%	2	4,5%		

Na seção Resultados, no 6º parágrafo, página 6:

Onde se lia:

“Quando agrupadas as questões dos cinco *cases*, verifica-se que houve diferença significativa entre o grupo de intervenção para as questões 5 e 6, sendo que a questão 5 avaliou as atitudes, e a questão 6, as percepções dos universitários (Tabela 4).”

Leia-se:

“Quando agrupadas as questões dos cinco *cases*, verifica-se que houve diferença significativa entre o grupo de intervenção para a questão 5, que avaliou as atitudes dos universitários (Tabela 4).”

Na seção Resultados, na Tabela 4, página 7:

Onde se lia:

**Table:** 

**Variável**	**Grupo**	**Média**	DP*	***p* -valor** ^†^
Q ^‡^ 6% acertos	Intervenção	79,091	4,2640	0.035 ^§^
	Controle	89,091	21,1365	
Questão total acertos %	Intervenção	75,758	6,6812	0,177 ^§^
	Controle	72,424	9,2110	

Leia-se:

**Table:** 

**Variável**	**Grupo**	**Média**	DP*	***p* -valor** ^†^
Q ^‡^ 6% acertos	Intervenção	97,3	12,79	0,128
	Controle	89,1	21,14	
Questão total acertos %	Intervenção	78,8	7,38	0,015 ^§^
	Controle	72,4	9,18	

Na seção Destaques, na página 1, foi excluída a seguinte informação: “**(2)** Aumento da percepção sobre os direitos das pessoas idosas em espaços públicos”.

Na seção Resumo, em Resultados, na página 1, foi excluída a seguinte informação: “e percepções...”.

Na seção Resultados, no 5º parágrafo, na página 6, foi excluída a seguinte informação: “no que se refere à questão 6 −”*Você considera que situações como esta podem ser prevenidas?*” −, que avaliou a percepção do universitário em relação à prevenção de uma situação de violência contra a pessoa idosa, no que tange aos seus direitos na sociedade.”.

Na seção Discussão, no 1º parágrafo (linha 6), página 7, foi excluída a seguinte informação: “e percepções”.

Na seção Discussão, página 8, foi excluído por completo o 10º parágrafo.

Na seção Discussão, no último parágrafo (linha 4), página 9, foi excluída a seguinte informação: “e percepções”.

Na seção Conclusão, no 1º parágrafo (linha 3), página 9, foi excluída a seguinte informação: “e no aumento da percepção”.

# Correction: Fe de Erratas: Efecto de una intervención educativa para estudiantes universitarios sobre la violencia contra las personas mayores utilizando la gamificación: ensayo clínico no aleatorizado

**DOI:** 10.1590/1518-8345.0000.4740

**Published:** 2025

En el artículo “Efecto de una intervención educativa para estudiantes universitarios sobre la violencia contra las personas mayores utilizando la gamificación: ensayo clínico no aleatorizado”, con número DOI: 10.1590/1518-8345.7874.4661, publicado en la Rev. Latino-Am. Enfermagem, 2025;33:e4661:

En la sección Resultados, en el 3º párrafo, página 6:

Donde se leía:

“En los *Cases* 2, 3, y 5”

Leer:

“En los *Cases* 2, 3, 4 y 5”

En la sección Resultados, en el 4º párrafo, página 6:

Donde se leía:

“La Tabla 3 muestra que, en el *Case* 4, hubo una diferencia estadísticamente significativa entre los grupos de intervención y de control”

Leer:

“La Tabla 3 muestra que, en el *Case* 4, no hubo diferencia estadísticamente significativa entre los grupos de intervención y de control”

En la sección Resultados, en la Tabla 3, página 6:

Donde se leía:

**Table:** 

		**Grupo**				**Total**		valor *p* a*	**valor *p* b** ^†^
		**Intervención (n=22)**		**Control (n=22)**					
		N	**%**	**N**	**%**	**N**	**%**		
Case 4 **Q** ^§^ **6**	Si	1	4,5%	20	90,9%	21	47,7%	<0,001 ^‡^	<0,001 ^||^
	No	21	95,5%	0	0,0%	21	47,7%		
	No puedo asegurarlo	0	0,0%	2	9,1%	2	4,5%		

Leer:

**Table:** 

		**Grupo**				**Total**		valor *p* a*	**valor *p* b** ^†^
		**Intervención (n=22)**		**Control (n=22)**					
		N	**%**	**N**	**%**	**N**	**%**		
Case 4 **Q** ^§^ **6**	Si	21	95,5%	20	90,9%	41	93,2%	<0,001 ^‡^	<0,220
	No	1	4,5%	0	0,0%	1	2,3%		
	No puedo asegurarlo	0	0,0%	2	9,1%	2	4,5%		

En la sección Resultados, en el 5º párrafo, página 6:

Donde se leía:

“Cuando se agrupan las preguntas de los cinco *cases*, se observa que había una diferencia significativa entre el grupo de intervención para las preguntas 5 y 6, en las que la pregunta 5 evaluaba las actitudes y la pregunta 6 las percepciones de los estudiantes universitarios (Tabla 4).”

Leer:

“Cuando se agrupan las preguntas de los cinco *cases*, se observa que había una diferencia significativa entre el grupo de intervención para la pregunta 5, que evaluaba las actitudes de los estudiantes universitarios (Tabla 4).”

En la sección Resultados, en la Tabla 4, página 7:

Donde se leía:

**Table:** 

**Variable**	**Grupo**	**Media**	DE*	**valor** ***p*** ^†^
**Q** ^‡^ **6% aciertos**	Intervención	79,091	4,2640	0.035 ^§^
	Control	89,091	21,1365	
**Total de aciertos Cuestiones%**	Intervención	75,758	6,6812	0,177 ^§^
	Control	72,424	9,2110	

Leer:

**Table:** 

**Variable**	**Grupo**	**Media**	DE*	**valor** ***p*** ^†^
**Q** ^‡^ **6% aciertos**	Intervención	97,3	12,79	0,128
	Control	89,1	21,14	
**Total de aciertos Cuestiones%**	Intervención	78,8	7,38	0,015 ^§^
	Control	72,4	9,18	

En la sección Destacados, página 1, se ha eliminado la siguiente información: “**(2)** Hay una mayor concienciación sobre los derechos de las personas mayores en los espacios públicos.”.

En la sección Resumen, en Resultados, página 1, se ha eliminado la siguiente información: “y percepciones”.

En la sección Resultados, en el 4º párrafo, página 6, se ha eliminado la siguiente información: “con respecto a la pregunta 6 −”*¿Cree que se pueden prevenir situaciones como ésta?*” − que evaluaba la percepción de los estudiantes universitarios sobre cómo prevenir la violencia contra las personas mayores en lo que se refiere a sus derechos en la sociedad.”.

En la sección Discusión, en el primer párrafo (línea 7), página 7, se ha eliminado la siguiente información: “y percepciones”.

En la sección Discusión, página 8, se eliminó por completo el noveno párrafo.

En la sección Discusión, en el último párrafo (línea 4), página 9, se ha eliminado la siguiente información: “y percepciones”.

En la sección Conclusión, en el primer párrafo (línea 3), página 9, se ha eliminado la siguiente información: “y aumentar la concienciación”.
